# Fas signaling-mediated T_H_9 cell differentiation favors bowel inflammation and antitumor functions

**DOI:** 10.1038/s41467-019-10889-4

**Published:** 2019-07-02

**Authors:** Yingying Shen, Zhengbo Song, Xinliang Lu, Zeyu Ma, Chaojie Lu, Bei Zhang, Yinghu Chen, Meng Duan, Lionel Apetoh, Xu Li, Jufeng Guo, Ying Miao, Gensheng Zhang, Diya Yang, Zhijian Cai, Jianli Wang

**Affiliations:** 1 Institute of Immunology and Bone Marrow Transplantation Center of the First Affiliated Hospital, Zhejiang University School of Medicine, 310058 Hangzhou, China; 2 Institute of Hematology, Zhejiang University and Zhejiang Engineering Laboratory for Stem Cell and Immunotherapy, 310003 Hangzhou, China; 3 Institute of Immunology and Department of Orthopaedics of the Second Affiliated Hospital, Zhejiang University School of Medicine, 310058 Hangzhou, China; 4 Department of Medical Oncology, Zhejiang Cancer Hospital, 310022 Hangzhou, China; 5 Division of Infection Disease, Zhejiang Key Laboratory for Neonatal Diseases, Children’s Hospital, Zhejiang University School of Medicine, 310006 Hangzhou, China; 6 Chronic Disease Research Institute, School of Public Health, School of Medicine, Zhejiang University, 310058 Hangzhou, China; 7 INSERM, U866 Dijon, France; 8 Faculté de Médecine, Université de Bourgogne, Dijon, 21000 France; 9 School of Life Science, Westlake University, 310024 Hangzhou, China; 10 Department of Breast Surgery, Affiliated Hangzhou First People’s Hospital, Zhejiang University School of Medicine, 310006 Hangzhou, China; 11 Clinical Trial Center, Qingdao Municipal Hospital, 266011 Qingdao, China; 12 Department of Critical Care Medicine, Second Affiliated Hospital, Zhejiang University School of Medicine, 310009 Hangzhou, China; 13 Xinyuan Institute of Medicine and Biotechnology, School of Life Sciences, Zhejiang Sci-Tech University, 310018 Hangzhou, China

**Keywords:** Cancer immunotherapy, Lymphocyte differentiation, CD4-positive T cells, Signal transduction

## Abstract

Fas induces apoptosis in activated T cell to maintain immune homeostasis, but the effects of non-apoptotic Fas signaling on T cells remain unclear. Here we show that Fas promotes T_H_9 cell differentiation by activating NF-κB via Ca^2+^-dependent PKC-β activation. In addition, PKC-β also phosphorylates p38 to inactivate NFAT1 and reduce NFAT1-NF-κB synergy to promote the Fas^-^induced T_H_9 transcription program. Fas ligation exacerbates inflammatory bowel disease by increasing T_H_9 cell differentiation, and promotes antitumor activity in p38 inhibitor-treated T_H_9 cells. Furthermore, low-dose p38 inhibitor suppresses tumor growth without inducing systemic adverse effects. In patients with tumor, relatively high T_H_9 cell numbers are associated with good prognosis. Our study thus implicates Fas in CD4^+^ T cells as a target for inflammatory bowel disease therapy. Furthermore, simultaneous Fas ligation and low-dose p38 inhibition may be an effective approach for T_H_9 cell induction and cancer therapy.

## Abstract (web summary)

Fas signalling induces apoptosis of activated T cells to maintain immune homeostasis. Here the authors show that Fas also induces PKC-β activation to promote NF-κB-mediated T_H_9 cell differentiation, while p38 activation by PKC-β antagonizes this effect, thereby supporting a synergy between p38 inhibitor and Fas for T_H_9 differentiation.

## Introduction

Receiving specific patterns of cytokine signaling during activation, CD4^+^ T cells will differentiate into different effector T cell subsets. Interleukin-12 (IL-12) promotes T helper type 1 (T_H_1) cell differentiation. IL-4 promotes T_H_2 cell differentiation. Transforming growth factor-β1 (TGF-β1) and IL-6 stimulate T_H_17 cell differentiation, while TGF-β1 alone drives T regulatory cell (Treg) differentiation^1–3^. IL-9-producing T_H_9 cells were first identified by reprogramming T_H_2 cells with TGF-β1 or Foxp3^+^ Tregs with IL-4^4,4^. Currently, it is generally accepted that IL-4 and TGF-β1 can induce T_H_9 cell differentiation^6^. T_H_9 cells are reported to exacerbate autoimmune and allergic diseases^7–10^. They also exhibit robust antitumor activity superior to that of T_H_1 and T_H_17 cells^11,12^. A recent publication demonstrated that one transfer of T_H_9 cells is sufficient to eradicate advanced tumors^13^, indicating the promise of T_H_9 cells in adoptive cancer therapy. Therefore, it is necessary to further elucidate the underlying mechanism of T_H_9 cell differentiation.

Accumulating evidence has unveiled the regulation of T_H_9 cell differentiation at the molecular level. CD4^+^ T cells with interferon-regulatory factor 4 (IRF4), GATA-3, or STAT6 deficiency fail to develop into T_H_9 cells^5,8,14^, indicating the essential roles of these transcription factors (TFs) in T_H_9 cell differentiation. PU.1 is also required for the development of T_H_9 cells^9^. The cooperative signaling among the TGF-β1-activated kinase TAK1, STAT5, Notch, Smad, and RBP-Jκ participates in T_H_9 cell differentiation^15,16^. OX40 and glucocorticoid-induced tumor necrosis factor receptor (TNF-R)-related protein, two members of the TNF-R superfamily, induce T_H_9 cells by activating the nuclear factor-κB (NF-κB) pathway^17,18^. Moreover, DR3, another member of the TNF-R family, enhances T_H_9 cell differentiation through a STAT5-dependent mechanism^19^. Whether other TNF-R family molecules regulate T_H_9 cell differentiation has yet to be explored.

Fas, a member of the TNF-R family, plays a critical role in programmed cell death^20^. Activation-induced cell death (AICD), mediated by the interaction of Fas and Fas ligand (FasL), is important in maintaining T cell homeostasis^21^. Fas is also necessary for T cell proliferation and activation^22,23^. A recent publication demonstrated that Fas promotes T_H_17 cell differentiation and inhibits T_H_1 cell development^24^. However, whether non-apoptotic Fas signaling participates in regulating T_H_9 cell differentiation remains unknown.

Here, we demonstrate that Fas activates protein kinase Cβ (PKCβ) in a Ca^2+^-dependent manner. PKCβ then induces NF-κB activation and, in cooperation with NFAT1, NF-κB-mediated T_H_9 cell differentiation. By contrast, PKCβ-activated p38 inactivates NFAT1, thereby limiting Fas-mediated T_H_9 cell differentiation. Fas ligation-induced T_H_9 cells (FasL-T_H_9) excerbates inflammatory bowel disease (IBD). In parallel, low-dose p38 inhibitor restores Fas-mediated T_H_9 cell induction in vitro and in vivo, and greatly suppresses tumor progression via IL-9 without inducing systemic adverse effects. Thus, our study reveals crucial functions of Fas-induced non-apoptotic signaling in T_H_9 cell induction, and may have important clinical implications in autoimmune disease and cancer therapy.

## Results

### Fas signaling promotes T_H_9 cell differentiation in vitro

**Fig. 1:**
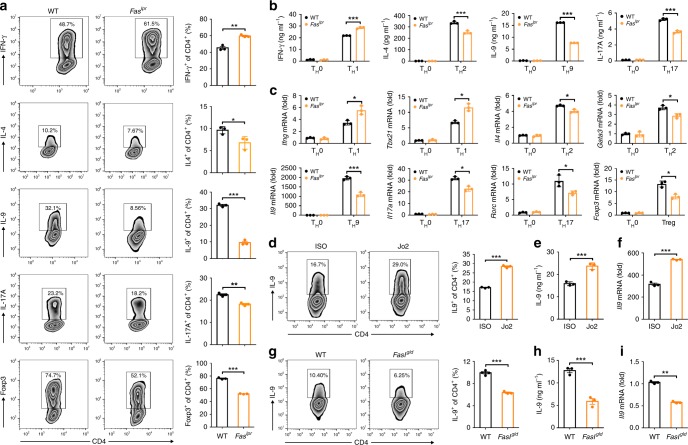
Fas signaling promotes T helper type 9 (T_H_9) cell differentiation in vitro. **a**–**c** Naiïve CD4^+^CD62L^hi^CD44^lo^ T cells were sorted from wild-ype (WT) and *Fas*^*lpr*^ mice and differentiated into T_H_0, T_H_1, T_H_2, T_H_9, and T_H_17 cells and T regulatory cells (Tregs) in the presence of plate-bound anti-CD3 and anti-CD28 for 3–4 days. Flow cytometric analysis of the frequencies of IFN-γ^+^, IL-4^+^ (interleukin-4^+^), IL-9^+^, IL-17A^+^, and Foxp3^+^ cells among CD4^+^ T cells (left) and the corresponding statistical analysis (right) on day 4 (**a**); enzyme-linked immunosorbent assay (ELISA) measurements of the IFN-γ, IL-4, IL-9 and IL-17A levels in supernatants from the T_H_0, T_H_1, T_H_2, T_H_9, and T_H_17 cells on day 3 (**b**); and real-time PCR analysis of the expression of the indicated genes in the T_H_0, T_H_1, T_H_2, T_H_9, and T_H_17 cells and Tregs on day 3 (**c**). **d**–**f** Naive CD4^+^CD62L^hi^CD44^lo^ T cells were sorted from WT mice and differentiated into T_H_9 cells with 10 μg ml^−1^ isotype control antibodies (ISO) or anti-Fas antibodies (Jo2) for 3–4 days. Flow cytometric analysis of the frequency of IL-9^+^ cells among the CD4^+^ T cells (left) and the corresponding statistical analysis (right) (**d**), ELISA measurement of the IL-9 cytokine levels in the supernatants of the T_H_9 cells (**e**), and real-time PCR analysis of *Il9* gene expression in the T_H_9 cells (**f**). **g**–**i** Naive CD4^+^CD62L^hi^CD44^lo^ T cells were sorted from WT and *Fas*^*gld*^ mice and differentiated into T_H_9 cells for 3–4 days. Flow cytometric analysis of the frequency of IL-9^+^ cells among the CD4^+^ T cells (left) and the corresponding statistical analysis (right) (**g**), ELISA measurement of the IL-9 cytokine levels in the supernatants of the T_H_9 cells (**h**), and real-time PCR analysis of *Il9* gene expression in the T_H_9 cells (**i**). *P < 0.05, **P < 0.01, and ***P < 0.001 (unpaired Student’s *t* test). Representative results from three independent experiments are shown (mean and s.d.) (*n* = 3)

To determine the role of Fas signaling in T_H_9 cell differentiation, we differentiated naive CD4^+^ T cells from wild-type (WT) and *Fas*^*lpr*^ mice (termed WT or *Fas*^*lpr*^ CD4^+^ T cells, respectively) into T_H_1, T_H_2, T_H_9, and T_H_17 cells and Tregs in vitro. The *Fas*^*lpr*^ CD4^+^ T cells generated substantially lower levels of IL-9-producing cells and IL-9 protein than did the WT CD4^+^ T cells (Fig. 1a, b). Consistent with a previous publication^24^, increased T_H_1 and decreased T_H_2 and T_H_17 cell differentiation could be observed with the *Fas*^*lpr*^ CD4^+^ T cells (Fig. 1a, b). However, Treg differentiation was significantly inhibited in our polarization systems (Fig. 1a). Changes in signature cytokines or TF messenger RNA (mRNA) levels between the WT and *Fas*^*lpr*^ CD4^+^ T cells also confirmed these results (Fig. 1c). T_H_9 cells differentiated from the WT or *Fas*^*lpr*^ CD4^+^ T cells (termed WT-T_H_9 and *Fas*^*lpr*^-T_H_9, respectively) expressed high levels of the genes encoding the T_H_9-related TFs PU.1 (*Spi1*) and IRF4 (*Irf4*) (Supplementary Fig. 1a). Nevertheless, the gene levels of the T_H_1-, T_H_2-, T_H_17-, and Treg-related TFs T-bet (*Tbx21*), GATA-3 (*Gata3*), RORγt (*Rorc*), and Foxp3 (*Foxp3*), respectively, and the corresponding signature cytokines IFN-γ (*Ifng*), IL-4 (*Il4*), and IL-17A (*Il17a*) were very low in these cells (Supplementary Fig. 1a). These results demonstrated that Fas signaling enhanced, but did not skew, T_H_9 cell differentiation. T_H_9 differentiation in WT CD4^+^ T cells with Fas knockdown was also blunted (Supplementary Fig. 1b–d), excluding the possibility that the decreased T_H_9 cell-polarizing ability resulted from developmental defect in the *Fas*^*lpr*^ CD4^+^ T cells. To further confirm the role of Fas signaling in T_H_9 cell differentiation, we ligated Fas on WT CD4^+^ T cells with anti-Fas (Jo2) in vitro and found that Jo2 markedly increased the frequency of IL-9-producing T cells and the IL-9 protein and mRNA levels (Fig. 1d–f). As Fas signaling mediates T cell apoptosis^25,26^, we detected the apoptosis of T_H_9 cells with or without Jo2 stimulation. We found that Jo2 did not affect T_H_9 cell apoptosis (Supplementary Fig. 1e). Moreover, Jo2 did not alter T_H_9 cell proliferation (Supplementary Fig. 1f). Fas signaling-induced AICD is the essential mechanism that maintains T cell homeostasis^21^. To assess AICD in FasL-T_H_9, T_H_9 cells induced by conventional or Fas ligation methods were restimulated with anti-CD3 and anti-CD28. Compared with the conventionally differentiated T_H_9 cells (cT_H_9), the FasL-T_H_9 showed increased AICD (Supplementary Fig. 1g). However, the FasL-T_H_9 secreted more IL-9 than the cT_H_9 (Supplementary Fig. 1h). These results further proved that Fas signaling promotes induction of IL-9-producing T cells.

To test whether autoactivated Fas signaling can increase IL-9-producing T cell numbers, we detected *Fas* and *Fasl* mRNA levels in T_H_ cell subsets and found that T_H_9 cells had lower *Fas* but higher *Fasl* gene expression than the other T_H_ subsets (Supplementary Fig. 1i, j). The ratio of the *Fas* gene level to the *Fasl* gene level was highest in the T_H_9 cells (Supplementary Fig. 1k), suggesting that autoactivated Fas signaling may play an important role in the induction of IL-9-producing T cells. Then, we differentiated naive CD4^+^ T cells from WT and *Fasl*^*gld*^ mice into T_H_9 cells in vitro. FasL deficiency greatly inhibited the induction of IL-9-producing T cells (Fig. 1g–i), and a similar inhibitory effect was also achieved with FasL blocking antibodies (anti-FasL) (Supplementary Fig. 1l). Transfection of a FasL-expressing vector but not an empty vector (EV) rescued the decreased induction of IL-9-producing T cells within the CD4^+^ T cells from *Fasl*^*gld*^ mice (Supplementary Fig. 1m). These findings indicated that autoactivated Fas signaling reinforces the induction of IL-9-producing T cells in vitro.

### Fas signaling activates genes related to T_H_9 cell functions

**Fig. 2:**
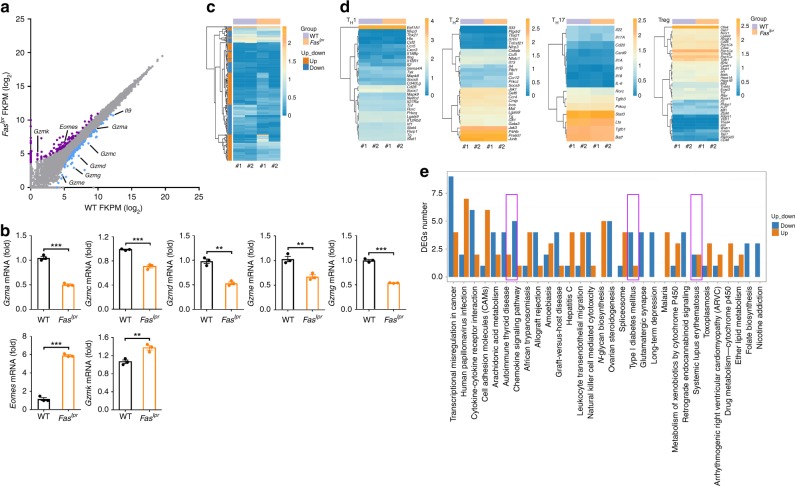
Fas signaling activates genes related to T helper type 9 (T_H_9) cell functions. **a** Scatterplot of RNA-sequencing data showing genes with expression upregulated (purple dots) or downregulated (blue dots) by at least two-fold in *Fas*^*lpr*^-T_H_9 cells relative to wild-type (WT)-T_H_9 cells and genes with similar expression in *Fas*^*lpr*^-T_H_9 and WT-T_H_9 cells (gray dots). **b** Real-time PCR analysis of the expression of the indicated genes in T_H_9 cells differentiated from WT or *Fas*^*lpr*^ CD4^+^ T cells. **c** Heat map of genes expressed in *Fas*^*lpr*^-T_H_9 and WT-T_H_9 cells. **d** Heat map of T_H_1 cell-, T_H_2 cell-, T_H_17 cell-, and Treg-related genes expressed in *Fas*^*lpr*^-T_H_9 and WT-T_H_9 cell. **e** Differentially expressed gene (DEG) results for the most highly enriched pathways. Pathways referring to T_H_9 cell functions are boxed. FKPM, fragments per kilobase of exon per million fragments mapped. **P < 0.01 and ***P < 0.001 (unpaired Student’s *t* test). Representative results from three independent experiments are shown (mean and s.d.) (*n* = 3)

To better understand the effects of Fas signaling on the T_H_9 cell program, we performed RNA-sequencing (RNA-seq) analysis of WT-T_H_9 and *Fas*^*lpr*^-T_H_9. There were 204 differentially expressed genes (DEGs) between the WT-T_H_9 and *Fas*^*lpr*^-T_H_9 (Supplementary Data 1). Among these DEGs, 84 genes had upregulated expression, and 120 genes had downregulated expression in the *Fas*^*lpr*^-T_H_9 (Fig. 2a). *Il9* was one of the most highly downregulated genes in the *Fas*^*lpr*^-T_H_9 (Fig. 2a). *Gzm* genes have been reported to have increased expression in T_H_9 cells and contribute to the antitumor activity of T_H_9 cells^13^. We found that *Gzma*, *Gzmc*, *Gzmd*, *Gzme*, and *Gzmg* were downregulated, but the master regulator of granzyme *Eomes*^27^ and *Gzmk* were upregulated in the *Fas*^*lpr*^-T_H_9 (Fig. 2a), which were confirmed by real-time PCR (Fig. 2b), suggesting *Eomes*-independent regulation of granzyme in T_H_9 cells. A heat map obtained by unsupervised hierarchical clustering showed that the WT-T_H_9 and *Fas*^*lpr*^-T_H_9 converged in the same cluster and showed overlapping gene expression (Fig. 2c). Moreover, T_H_1 cell-, T_H_2 cell-, T_H_17 cell-, and Treg-related genes showed no obvious differences between the WT-T_H_9 and *Fas*^*lpr*^-T_H_9 (Fig. 2d). These results indicated that Fas deficiency does not globally affect T_H_9 cell differentiation. T_H_9 cells play a key role in the progression of autoimmune diseases^28^. DEG results from an analysis of the most highly enriched pathways revealed that genes related to autoimmune thyroid disease, type I diabetes mellitus, and systemic lupus erythematosus were downregulated in the *Fas*^*lpr*^-T_H_9 (Fig. 2e). Therefore, RNA-seq analysis demonstrated that Fas signaling contributes to the gene activation, and is responsible for T_H_9 cell-specific functions.

### Fas signaling induces T_H_9 cells by activating NF-κB

**Fig. 3:**
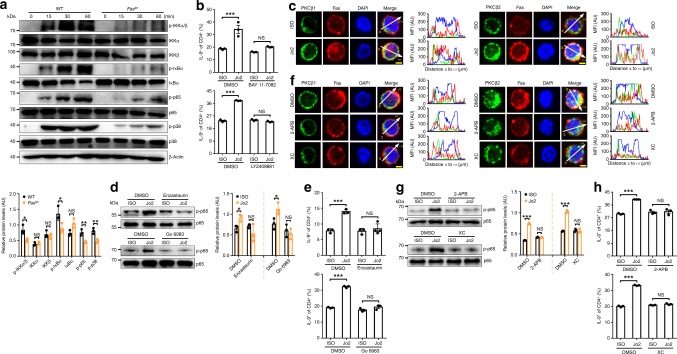
Fas signaling induces T helper type 9 (T_H_9) cell generation by activating nuclear factor-κB (NF-κB). **a** Western blotting analysis of the indicated proteins in wild-type (WT) or *Fas*^*lpr*^ CD4^+^ T cells under T_H_9-skewing conditions at the indicated time points (top), and statistical analysis of the relative protein levels at the 60 min (bottom). **b** Flow cytometric analysis of the frequency of IL-9^+^ (interleukin-9^+^) cells among CD4^+^ T cells after the stimulation of naive CD4^+^ T cells with 10 μg ml^−1^ ISO or Jo2 with or without 0.4 μM IκBα inhibitor BAY 11-7082 or 0.5 μM IKK2 inhibitor LY2409881 under T_H_9-skewing conditions for 4 days. **c** Immunofluorescence staining of protein kinase Cβ (PKCβ1) or PKCβ2 with Fas in naive CD4^+^ T cells stimulated with 10 μg ml^−1^ ISO or Jo2 for 15 min under T_H_9-skewing conditions. **d** Western blotting analysis of p-p65 protein levels in naive CD4^+^ T cells stimulated with 10 μg ml^−1^ ISO or Jo2 for 15 min with or without 0.5 μM PKCβ inhibitor enzastaurin or 0.01 μM pan-PKC inhibitor Go 6983 under T_H_9-skewing conditions (left), and statistical analysis of the relative protein levels (right). **e** Flow cytometric analysis of the frequency of IL-9^+^ cells among CD4^+^ T cells after the stimulation of naive CD4^+^ T cells with 10 μg ml^−1^ ISO or Jo2 with or without 0.5 μM enzastaurin or 0.01 μM Go 6983 under T_H_9-skewing conditions for 4 days. **f**–**h** Immunofluorescence staining of PKCβ1 or PKCβ2 with Fas (**f**), western blotting analysis of p-p65 protein levels (left) and statistical analysis of the relative protein levels (right) (**g**), and flow cytometric analysis of the frequency of IL-9^+^ cells (**h**) in the naive CD4^+^ T cells stimulated with 10 μg ml^−1^ Jo2 with or without 10 μM inositol 1,4,5-trisphosphate-induced Ca^2+^ release inhibitor, 2-aminoethoxydiphenyl borate (2-APB) or xestospongin C (XC) for 15 min (**f**, **g**) or 4 days (**h**) under T_H_9-skewing conditions. Scale bar = 2 μm. AU, arbitrary units; NS, not significant; *P < 0.05, **P < 0.01, ***P < 0.001 (unpaired Student’s *t* test). Representative results from three independent experiments are shown (mean and s.d.) (*n* = 3). Relative protein levels = the indicated protein gray value/β-actin (**a**) or p65 (**d**, **g**) gray value

To examine how Fas signaling induces T_H_9 cells, we first investigated the differentiation of FasL-T_H_9 in the presence of the pancaspase inhibitor z-VAD-fmk^29^ and found that z-VAD-fmk did not alter the Jo2-mediated increase in IL-9-producing T cells (Supplementary Fig. 2a), indicating caspase-independent induction of T_H_9 cells. Since Fas defects inhibit T_H_9 cell and Treg differentiation, and IL-2 is critical for both cell differentiation^30,31^, we next tested the role of IL-2 in Fas-mediated T_H_9 cell differentiation. We found that *Il2* mRNA levels were not different between WT-T_H_9 and *Fas*^*lpr*^-T_H_9 (Supplementary Fig. 2b). Moreover, neither the addition of exogenous IL-2 nor neutralization of endogenous IL-2 rescued differentiation inferiority of *Fas*^*lpr*^-T_H_9 (Supplementary Fig. 2c, d), indicating IL-2-independent increase of T_H_9 cell differentiation by Fas. STAT1, STAT3, STAT5, STAT6, IRF4, PU.1, Gata3, NF-κB, and Akt are all involved in T_H_9 cell differentiation^6,12,18,32,33^. Mitogen-activated protein kinases also participate in IL-9 expression^34^. We detected the activation and total protein levels of these TFs and kinases in WT and *Fas*^*lpr*^ CD4^+^ T cells cultured under T_H_9-skewing conditions for 15 min or 24 h and found that the activation of p65 and p38 was obviously inhibited in the *Fas*^*lpr*^ CD4^+^ T cells (Supplementary Fig. 2e, f). In contrast, Fas ligation markedly induced the activation of p65 and p38 (Supplementary Fig. 2e, f). Furthermore, the activation of IKKα, IKKβ, IκBα, p65, and p38 markedly decreased in the *Fas*^*lpr*^ CD4^+^ T cells at different time points (Fig. 3a), indicating Fas signaling-dependent activation of the NF-κB and p38 pathways in differentiating T_H_9 cells. To test the role of the NF-κB pathways in Fas-mediated T_H_9 cell differentiation, we treated CD4^+^ T cells with the IκBα phosphorylation inhibitor BAY 11-7082^35^ or the IKK2 inhibitor LY2409881^36^ before inducing polarization. Both BAY 11-7082 and LY2409881 completely abolished the differentiation superiority of FasL-T_H_9 (Fig. 3b), indicating that Fas-induced IL-9-producing T cell generation is NF-κB dependent.

PKC has been reported to participate in the activation of NF-κB^37^. There are three major groups of PKC isoforms. These groups include classic PKCs (PKCα, PKCβ1, PKCβ2, and PKCγ), novel PKCs (PKCδ, PKCε, PKCζ, PKCμ, and PKCθ), and atypical PKCs (PKCζ and PKCι/λ). Fas is known to play a role in PKCβ2 activation^38^. Next, we examined whether PKC is responsible for Fas-induced NF-κB activation. We found that Fas ligation obviously increased the recruitment of PKCβ2 to the plasma membrane and its colocalization with Fas (Fig. 3c). Increased PKCβ1 recruitment to the plasma membrane and colocalization with Fas were also observed after Fas ligation (Fig. 3c). The selective PKCβ inhibitor enzastaurin^39^ and the pan-PKC inhibitor Go 6983 both abrogated the Fas ligation-induced phosphorylation of p65 and increase in IL-9-producing T cells (Fig. 3d, e). These results demonstrated that PKCβ behaves as an activator of NF-κB after Fas ligation.

The activation of classic PKCs is Ca^2+^ dependent^40^. Jo2 stimulation evoked Ca^2+^ flux in WT CD4^+^ T cells under T_H_9-skewing conditions (Supplementary Fig. 2g). To define the function of Ca^2+^ in PKCβ activation, we pretreated cells with 2-aminoethoxydiphenyl borate (2-APB) or xestospongin C (XC), which are inhibitors of inositol 1,4,5-trisphosphate-induced Ca^2+^ release^41,42^, and found that both prevented PKCβ2 and PKCβ1 recruitment to the plasma membrane and colocalization with Fas in response to Fas ligation (Fig. 3f). Consistently, both inhibitors abolished the activation of p65 and enhancement of IL-9-producing T cell generation (Fig. 3g, h), suggesting that PKCβ activation by Fas ligation is Ca^2+^ dependent.

Fas can trigger Ca^2+^ signaling by activating phospholipase Cγ1 (PLCγ1)^43^. We observed reduced phosphorylated PLCγ1 level in *Fas*^*lpr*^ CD4^+^ T cells under T_H_9-skewing conditions (Supplementary Fig. 2h). Both U73122, a potent PLC inhibitor^44^, and manoalide, an irreversible PLC inhibitor^45^, abolished Fas ligation-induced increases in Ca^2+^ flux and IL-9-producing T cells (Supplementary Fig. 2i, j), suggesting that the increased Ca^2+^ response by Fas signaling is PLCγ1-dependent.

### Tyr224 and Tyr274 in Fas mediate PLCγ1 activation

Given that Fas has no enzymatic activity^46^, we questioned how PLCγ1 was activated. Zap-70 can interact with Fas and participate in the phosphorylation of PLCγ1 in T cells^47,48^. Fas ligation obviously increased the colocalization of Fas and Zap-70 and phosphorylated Zap-70 level under T_H_9-skewing conditions (Supplementary Fig. 3a, b). Knocking down Zap-70 expression markedly decreased the Fas ligation-induced phosphorylation of PLCγ1 and generation of IL-9-producing T cell (Supplementary Fig. 3c–f), indicating the Zap-70-dependent phosphorylation of PLCγ1.

There are four Tyr sites in the intracellular domain of Fas (Tyr189, Tyr224, Tyr274, and Tyr284). Tyr284, which is located in the YXXL motif of Fas, is necessary for the binding of Fas with Zap-70^47^. However, a Y284A mutation in Fas did not reduce the colocalization of Fas and Zap-70 (Supplementary Fig. 3g). However, the mutations Y224A and Y274A, but not the mutation Y189A, greatly decreased the colocalization of Fas and Zap-70 (Supplementary Fig. 3g). Consistently, the overexpression of unmutated Fas (Fas-WT) or Fas with the Y189A or Y284A mutation (Fas-Y189A, Fas-Y284A) but not Fas with the Y224A or Y274A mutation (Fas-Y224A, Fas-Y274A) significantly promoted the generation of IL-9-producing T cells from naive *Fas*^*lpr*^ CD4^+^ T cells (Supplementary Fig. 3h). These results demonstrated that both Tyr224 and Tyr274 in Fas are necessary for the Fas ligation-induced generation of IL-9-producing T cells.

### p38 inhibits T_H_9 cell generation by mediated Fas signaling

**Fig. 4:**
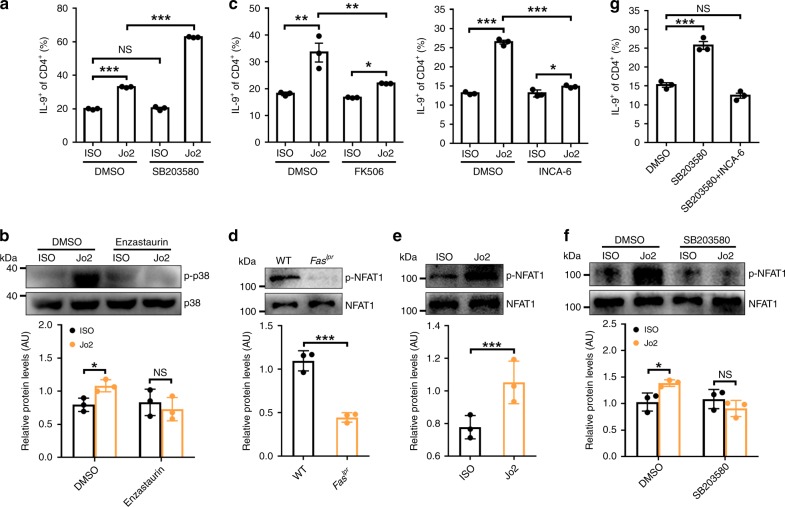
p38 inhibits T helper type 9 (T_H_9) cell generation by Fas signaling. **a** Flow cytometric analysis of IL-9^+^ (interleukin-9^+^) cell frequency among CD4^+^ T cells after naive CD4^+^ T cells were stimulated with 10 μg ml^−1^ ISO or Jo2 and with or without 0.4 μM p38 inhibitor SB203580 under T_H_9-skewing conditions for 4 days. **b** Western blotting analysis of p-p38 proteins in naive CD4^+^ T cells stimulated with 10 μg ml^−1^ ISO or Jo2 for 15 min and with or without 0.5 μM enzastaurin under T_H_9-skewing conditions (up), and statistical analysis of the relative protein levels (down). **c** Flow cytometric analysis of IL-9^+^ cell frequency among CD4^+^ T cells after naive CD4^+^ T cells were stimulated with 10 μg ml^−1^ ISO or Jo2 and with or without NFAT inhibitor (5 pM FK506 or 50 nM INCA-6) under T_H_9-skewing conditions for 4 days. **d**, **e** Western blotting analysis of p-NFAT1 proteins in WT or *Fas*^*lpr*^ CD4^+^ T cells (up) (**d**) or WT CD4^+^ T cells stimulated with 10 μg ml^−1^ ISO or Jo2 (**e**) under T_H_9-skewing conditions for 15 min (up), and statistical analysis of the relative protein levels (down) (**d**, **e**). **f** Western blotting analysis of p-NFAT1 proteins in WT CD4^+^ T cells stimulated with 10 μg ml^−1^ ISO or Jo2 and with or without 0.4 μM SB203580 under T_H_9-skewing conditions for 15 min (up) and statistical analysis of the relative protein levels (down). **g** Flow cytometric analysis of IL-9^+^ cell frequency among CD4^+^ T cells after naive CD4^+^ T cells were stimulated with 10 μg ml^−1^ Jo2 in the presence of 0.4 μM SB203580 or 50 nM INCA-6 plus 0.4 μM SB203580 under T_H_9-skewing conditions for 4 days. AU, arbitrary units; NS, not significant; *P < 0.05, **P < 0.01, and ***P < 0.001 (unpaired Student’s *t* test (**a**, **c**, **g**)). Representative results from three independent experiments are shown (mean and s.d.). Relative protein levels = the indicated protein gray values/p38 (**b**) or NFAT1 (**d**–**f**) gray values

Since Fas ligation obviously induced p38 activation (Supplementary Fig. 2e), we examined the role of p38 in the differentiation of FasL-T_H_9. Strikingly, Fas ligation induced a much higher frequency of IL-9-producing T cells in cells treated with the p38 inhibitor SB203580 than in untreated cells (Fig. 4a). However, SB203580 alone could not increase cT_H_9 (Fig. 4a). Additionally, knocking down p38α expression promoted the differentiation of FasL-T_H_9 but not cT_H_9 (Supplementary Fig. 4a, b). PKCβ can mediate p38 activation^49^. To elucidate whether PKCβ is involved in Fas ligation-induced p38 activation, we treated WT CD4^+^ T cells with enzastaurin and found no increased activation of p38 due to Fas ligation (Fig. 4b). These results demonstrated that PKCβ-activated p38 provides negative feedback in the differentiation of FasL-T_H_9.

NFAT1 cooperates with NF-κB to induce IL-9 transcription in CD4^+^ T cells^50^. p38 can inhibit NFAT signaling by mediating NFAT phosphorylation^51^. We hypothesized that p38 might inhibit the differentiation of FasL-T_H_9 by regulating NFAT1 phosphorylation. We first determined the role of NFAT in Fas signaling-induced IL-9-producing T cell generation and found that both FK506, an inhibitor of NFAT calcineurin inactivation^52^, and INCA-6, a potent and selective inhibitor of calcineurin-NFAT signaling^53^, partially inhibited the differentiation of FasL-T_H_9 (Fig. 4c). Neither inhibitor could restrain the Fas ligation-induced activation of NF-κB (Supplementary Fig. 4c). These results indicated that NFAT1 synergistically enhanced the NF-κB-mediated induction of IL-9-producing T cells by Fas ligation. NFAT1 phosphorylation was markedly reduced in *Fas*^*lpr*^ CD4^+^ T cells under T_H_9-skewing conditions (Fig. 4d), indicating NFAT1 activation. In contrast, Fas ligation increased NFAT1 phosphorylation in WT CD4^+^ T cells (Fig. 4e). Then, we examined the effect of p38 on NFAT1 phosphorylation and found that SB203580 treatment abolished the Fas ligation-induced increase in NFAT1 phosphorylation under T_H_9-skewing conditions (Fig. 4f). Furthermore, SB203580 treatment resulted in no difference in phosphorylated NFAT1 levels between *Fas*^*lpr*^ and WT CD4^+^ T cells under T_H_9-skewing conditions (Supplementary Fig. 4d). In the presence of INCA-6, SB203580 could no longer enhance the differentiation of FasL-T_H_9 (Fig. 4g). These results indicated that p38 limits the differentiation of FasL-T_H_9 by affecting NFAT1 phosphorylation.

### FasL-T_H_9 exacerbate murine IBD via IL-9

**Fig. 5:**
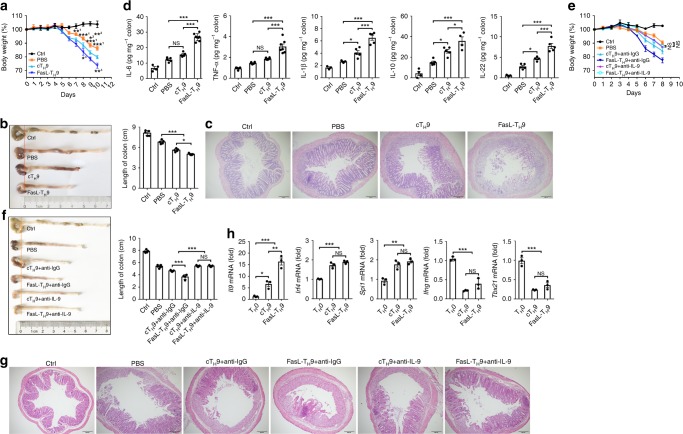
FasL-T_H_9 exacerbate murine inflammatory bowel disease (IBD) via interleukin-9 (IL-9). **a**–**d** Weight (*n* = 5) (**a**), colonic length (*n* = 5) (**b**), hematoxylin–eosin-stained colonic tissue sections (**c**), and enzyme-linked immunosorbent assay (ELISA) measurement of the indicated cytokines released by colonic tissue (*n* = 3–6) (**d**) of 2.5% dextran sulfate sodium salt (DSS) (w/v)-induced IBD mice that received an intravenous transfer of 2 × 10^6^ conventionally differentiated T_H_9 cells (cT_H_9) or Fas ligand (FasL)-T_H_9. **e**–**g** Weight (*n* = 5) (**e**), colonic length (*n* = 5) (**f**), and hematoxylin–eosin-stained of colonic tissue sections (**g**) of 2.5% DSS (w/v)-induced IBD mice that received an intravenous transfer of 2 × 10^6^ cT_H_9 or FasL-T_H_9 plus intravenous injection of 100 µg of anti-IgG- or anti-IL-9-neutralizing antibodies every other day. **h** Real-time PCR measurement of the indicated genes in CD45.1^+^ cT_H_9 or FasL-T_H_9 sorted from lamina propria lymphocytes (LPLs) isolated from 2.5% DSS (w/v)-induced CD45.2^+^ IBD mice that received an intravenous transfer of 1 × 10^7^ CD45.1^+^ cT_H_9 or FasL-T_H_9 for 5 days (*n* = 3). Scale bar = 200 μm. NS, not significant; *P < 0.05, **P < 0.01, and ***P < 0.001 (unpaired Student’s *t* test: **a**, **b** (right), **d**–**f** (right), and **h**). 1, compared with FasL-T_H_9; 2, compared with cT_H_9; and 3, compared with cT_H_9. Representative results from three independent experiments are shown (mean and s.d.)

IL-9 determines the pathogenesis of ulcerative colitis^10^. We tested the in vivo relevance of our observations in a murine IBD model. The transfer of either cT_H_9 or FasL-T_H_9 significantly increased weight loss and shortened colonic length in IBD mice (Fig. 5a, b). However, the ability of the FasL-T_H_9 to exacerbate IBD was stronger than that of the cT_H_9 (Fig. 5a, b). Histological analysis also revealed more leukocyte infiltration and more severe damage to glandular structures in the colonic tissue in the mice that received the transfer of FasL-T_H_9 (Fig. 5c). Moreover, FasL-T_H_9 caused colonic tissue to produce more IL-6, TNF, IL-1β, IL-10, and IL-22 (Fig. 5d). In contrast, compared with WT-T_H_9-treated mice, mice that received transfer of *Fas*^*lpr*^-T_H_9 showed reduced weight loss and colonic length shortening (Supplementary Fig. 5a, b). Histological assessment exhibited a similar trend (Supplementary Fig. 5c). These results indicated that Fas ligation exacerbated murine IBD. To test the effects of Fas signaling on T_H_9 cell apoptosis and proliferation in vivo, we transferred carboxyfluorescein succinimidyl ester (CFSE)-labeled WT-T_H_9 or *Fas*^*lpr*^-T_H_9 into IBD mice and evaluated apoptosis and proliferation in the CFSE-positive cells in the mesenteric lymph nodes. We found that there were no obvious differences in apoptosis or proliferation between the WT-T_H_9 and *Fas*^*lpr*^-T_H_9 (Supplementary Fig. 5d). These findings excluded the possibility that the apoptosis and proliferation of T_H_9 cells with Fas defects affect IBD pathogenicity.

To define the effect of IL-9 on T_H_9 cell-mediated IBD progression, we neutralized IL-9 with anti-IL-9 when cT_H_9 or FasL-T_H_9 was transferred. Both types of T_H_9 cells barely exacerbated murine IBD after IL-9 neutralization (Fig. 5e–g). We also detected similar effects of both types of T_H_9 cells on IBD *Il9r*^−/−^ mice (Supplementary Fig. 5e–g). These results demonstrated that the aggravation of murine IBD by FasL-T_H_9 depends on IL-9.

Published data suggest that in an inflammatory state in vivo, T_H_9 cells are unstable and begin to secrete IFN-γ^54^. Therefore, we examined the stability of T_H_9 cells in IBD mice in vivo by transferring Fas ligation-induced CD45.1^+^ T_H_9 cells into CD45.2^+^ IBD mice. We found that the CD45.1^+^ cells within the lamina propria lymphocytes (LPLs) maintained their expression of T_H_9-related genes (*Il9*, *Irf4*, and *Spi1*) and did not exhibit expression of T_H_1-related genes (*Ifng* and *Tbx21*) (Fig. 5h). These data indicated that FasL-T_H_9 are stable in IBD mice in vivo.

### Fas signaling relates to the antitumor activity of T_H_9 cells

T_H_9 cells are present in mice bearing melanoma tumor and exhibit prominent antitumor activity^11,55^. To dissect whether Fas signaling is related to T_H_9 cells in tumor-bearing mice, we injected B16F10 cells intravenously into WT mice and analyzed *Il9* mRNA levels in Fas-positive and Fas-negative CD4^+^ T cells. We found that the *Il9* mRNA levels in the Fas^+^CD4^+^ T cells were significantly higher than those in the Fas^−^CD4^+^ T cells (Supplementary Fig. 6a). In addition, we detected higher *Spi1* and *Irf4* mRNA levels in the Fas^+^CD4^+^ T cells than in the Fas^−^CD4^+^ T cells and similar *Tbx21*, *Gata3*, *Rorc*, and *Foxp3* mRNA levels between the two cell subsets (Supplementary Fig. 6a), further supporting the idea that Fas signaling dictates T_H_9 cell differentiation.

**Fig. 6:**
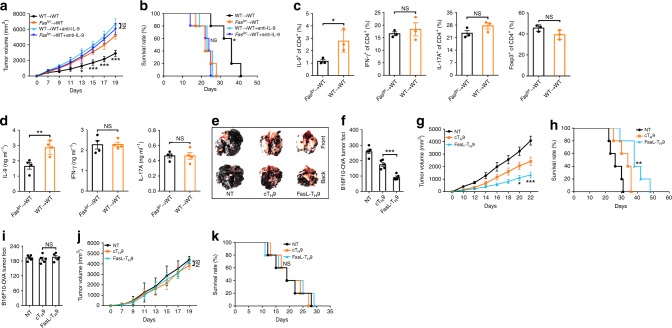
Fas signaling relates to the antitumor activity of T helper type 9 (T_H_9) cells. (**a**, **b**) Tumor growth (**a**) and survival (**b**) of irradiated WT mice reconstituted with bone marrow cells from wild-type (WT) or *Fas*^*lpr*^ mice for 2 months and then subcutaneously injected with LLC-OVA tumor cells with or without intravenous injection of 100 µg of anti-interleukin-9 (IL-9)-neutralizing antibodies every other day (*n* = 5). **c** Flow cytometric analysis of the frequencies of IL-9^+^, IFN-γ^+^, IL-17A^+^, and Foxp3^+^ cells among CD4^+^ T cells in the tumor-infiltrating lymphocytes (TILs) of the mice described in **a** 20 days after tumor inoculation (*n* = 3). (**d**) Enzyme-linked immunosorbent assay (ELISA) measurements of IL-9, IFN-γ, and IL-17A levels secreted by OVA_323–339_-stimulated TILs from the mice described in **a** 20 days after tumor inoculation (*n* = 3). **e**, **f** Representative lung appearance (**e**) and statistical analysis of the lung tumor foci (**f**) (*n* = 5) of WT mice 16 days after intravenous injection of B16F10-OVA melanoma cells with no transfer (NT) or transfer of OT-II cT_H_9 or FasL-T_H_9 1 and 6 days later. **g**, **h** Tumor growth (**g**) and survival (**h**) of WT mice that received a subcutaneous injection of B16F10-OVA cells followed by NT or the intravenous injection of 2 × 10^6^ OT-II cT_H_9 or FasL-T_H_9 1 and 6 days later (*n* = 5). **i** Lung tumor foci of *Il9r*^−*/−*^ mice 16 days after intravenous injection of B16F10-OVA melanoma cells with NT or transfer of OT-II cT_H_9 or FasL-T_H_9 1 and 6 days later (*n* = 5). **j**, **k** Tumor growth (**j**) and survival (**k**) of *Il9r*^*−/−*^ mice that received a subcutaneous injection of B16F10-OVA cells followed by NT or intravenous injection of 2 × 10^6^ OT-II cT_H_9 or FasL-T_H_9 1 and 6 days later (*n* = 5). NS, not significant; *P < 0.05, **P < 0.01, and ***P < 0.001 (unpaired Student’s *t* test: **a**, **c**, **d**, **f**, **g**, **i**, and **j**; log-rank test: **b**, **h**, **k**). Compared with the *Fas*^*lpr*^ → WT mice in **a**, **b**; compared with cT_H_9 in **g**, **h**, **j**, and **k**. Representative results from three independent experiments are shown (mean and s.d.)

To elucidate whether Fas signaling determines the antitumor effects of endogenous T_H_9 cells, we reconstituted irradiated WT mice with *Fas*^*lpr*^ (*Fas*^*lpr*^ → WT) or WT (WT → WT) bone marrow cells and established tumors with Lewis lung carcinoma cells expressing full-length ovalbumin (LLC-OVA) in these mice. We found that the tumor progression in the *Fas*^*lpr*^ → WT mice was superior to that in the WT → WT mice, which was IL-9 dependent (Fig. 6a, b). The tumor-infiltrating lymphocytes (TILs) in the WT → WT mice demonstrated an enhanced percentage of IL-9-producing CD4^+^ T cells but no increased percentages of IFN-γ- or IL-17A-producing CD4^+^ T cells or Tregs (Fig. 6c). Restimulation of TILs with OVA_323–339_ in vitro resulted in an evident increase in IL-9 protein expression, but not in IFN-γ or IL-17A protein expression, in the CD4^+^ T cells within the TILs of WT → WT mice (Fig. 6d). IL-9 is reported to exert an antitumor effect by activating CD8^+^ T cells^55^. We detected an increased frequency of IFN-γ-producing CD8^+^ T cells within the TILs of the WT → WT mice (Supplementary Fig. 6b). Moreover, restimulation of TILs with OVA_257–264_ in vitro led to a notable increase in IFN-γ protein levels in the CD8^+^ T cells with the TILs of the WT → WT mice (Supplementary Fig. 6c). These results suggested that Fas defects probably restrain antitumor immunity by suppressing IL-9-producing T cell generation.

To directly demonstrate that Fas signaling influences the antitumor properties of T_H_9 cells, we differentiated naive OT-II CD4^+^ T cells into T_H_9 cells with or without Jo2 stimulation. OT-II CD4^+^ T cells transgenically express a TCR recognizing an epitope of OVA_323–339_ in the context of I-Ab^12^. We intravenously transferred differentiated OT-II T_H_9 cells with OVA-expressing B16F10 cells (B16F10-OVA) into WT mice on the same day and found that FasL-T_H_9 exhibited stronger antitumor effects than cT_H_9 (Fig. 6e, f). FasL-T_H_9 also had superior antitumor effects on a B16F10-OVA tumor model established by subcutaneous inoculation (Fig. 6g, h). By using LLC-OVA, we obtained similar results (Supplementary Fig. 6d–f). To assess the role of IL-9 in the antitumor effects of FasL-T_H_9, we intravenously or subcutaneously challenged *Il9r*^*−/−*^ mice with B16F10-OVA tumor cells and found that neither FasL-T_H_9 nor cT_H_9 enhanced antitumor immunity (Fig. 6i–k). Collectively, these results demonstrated that FasL-T_H_9 enhance antitumor immunity via IL-9.

### p38 inhibitor exerts antitumor activity by inducing T_H_9

**Fig. 7:**
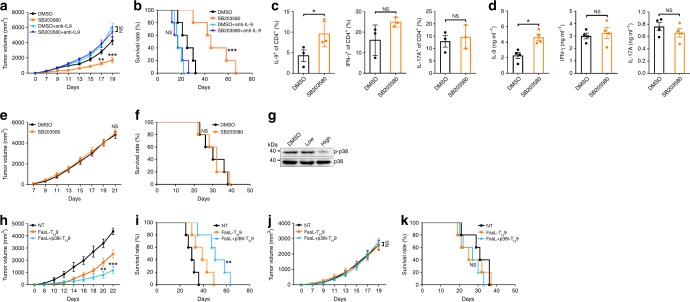
p38 inhibitor exerts antitumor activity by inducing T helper type 9 (T_H_9) cells. **a**, **b** Tumor growth (**a**) and survival (**b**) of wild-type (WT) mice that received a subcutaneous injection of LLC-OVA cells followed by an intraperitoneal injection of SB203580 (0.5 mg kg^−1^) with or without an intravenous injection of 100 µg of anti-IL-9-neutralizing antibodies every other day (*n* = 5). **c** Flow cytometric analysis of the frequencies of IL-9^+^ (interleukin-9^+^), IFN-γ^+^, and IL-17A^+^ cells among CD4^+^ T cells in the TILs of the mice described in **a** 20 days after tumor inoculation (*n* = 2–3). **d** Enzyme-linked immunosorbent assay (ELISA) measurements of IL-9, IFN-γ, and IL-17A levels secreted by OVA_323–339_-stimulated TILs from the mice described in **a** 20 days after tumor inoculation (*n* = 3). **e**, **f** Tumor growth (**e**) and survival (**f**) of *Fas*^*lpr*^ → WT mice that received a subcutaneous injection of LLC-OVA cells followed by an intraperitoneal injection of SB203580 (0.5 mg kg^−1^) every other day (*n* = 5). **g** Western blotting analysis of p-p38 expression in colonic tissues from mice that received intraperitoneal injection of low- (0.5 mg kg^−1^) or high-dose (10 mg kg^−1^) SB203580 every other day for a total of 11 injections. **h**–**k** Tumor growth (**h**) and survival (**i**) of WT mice or tumor growth (**j**) and survival (**k**) of *Il9r*^*−/−*^ mice that received a subcutaneous injection of B16F10-OVA cells followed by no transfer (NT) or intravenous injection of 2 × 10^6^ OT-II FasL-T_H_9 or FasL + p38i-T_H_9 1 and 6 days later (*n* = 5). NS, not significant; *P < 0.05, **P < 0.01, and ***P < 0.001 (unpaired Student’s *t* test: **a**, **c**–**e**, **h**, **j**; log-rank test: **b**, **f**, **i**, **k**). Compared with dimethyl sulfoxide (DMSO) in **a**, **b**; compared with FasL-T_H_9 in **h**–**k**. Representative results from three independent experiments are shown (mean and s.d.)

Given that a p38 inhibitor promoted Fas-induced IL-9-producing T cell generation, we investigated whether this p38 inhibitor could also exert antitumor effects dependent on T_H_9 cells. To test this, we first detected LLC-OVA tumor progression in WT mice with or without SB203580 treatment. Notable inhibition of tumor progression by SB203580 was observed, which was IL-9 dependent (Fig. 7a, b). The TILs from WT mice that received SB203580 treatment had a significantly higher frequency of IL-9-producing CD4^+^ T cells, but not the IFN-γ- or IL-17A-producing CD4^+^ T cells (Fig. 7c). Restimulation of the TILs with OVA_323–339_ in vitro induced increased production of the IL-9 protein, but not the IFN-γ or IL-17A protein, in the CD4^+^ T cells within the TILs of the WT mice that received SB203580 (Fig. 7d). Importantly, SB203580 could not inhibit tumor progression in *Fas*^*lpr*^ → WT mice, suggesting that SB203580 favored T_H_9 cell generation via Fas (Fig. 7e, f). Furthermore, SB203580 did not affect tumor growth in thymus-deficient nu/nu mice (Supplementary Fig. 7a), again highlighting the T cell-dependent antitumor effect. Usually, the dose of SB203580 used for in vivo treatment is 10 mg kg^−1^ (high)^56,57^, but the dose we used was 0.5 mg kg^−1^ (low). We dynamically monitored the SB203580 concentration in the plasma of mice that received low- or high-dose SB203580 treatment. The plasma SB203580 concentration reached a peak of 0.15 μg ml^−1^ at 1 h or of 19.46 μg ml^−1^ at 0.5 h in the mice receiving low- or high-dose SB203580 treatment, respectively (Supplementary Fig. 7b). At a dose of 20 μg ml^−1^, but not a dose of 0.15 μg ml^−1^, SB203580 significantly inhibited LLC-OVA cell viability in vitro (Supplementary Fig. 7c). These data further supported the idea of a T_H_9 cell-dependent antitumor effect of low-dose SB203580.

To evaluate the safety of systemic treatment with low- or high-dose SB203580, we compared the weights, and liver and renal functions of mice that received low-dose SB203580 treatment with those of mice that received high-dose SB203580 treatment. The two groups of mice showed no obvious differences in weight loss or impaired liver and renal functions (Supplementary Fig. 7d, e). Consistently, no pathological injury to the heart or other organs was found in either mouse group (Supplementary Fig. 7f). However, a decrease in the phosphorylated p38 level could be detected only in colonic tissues from the mice receiving high-dose SB203580 treatment (Fig. 7g), suggesting the possibility of systemic inhibition of the p38 pathway. Altogether, our data indicated that low-dose p38 inhibitor treatment restricts tumor progression by enhancing generation of IL-9-producing cells without systemic toxicity in vivo.

Since Fas ligation combined with the p38 inhibitor induced more IL-9-producing T cells, we hypothesized that Fas ligation plus a p38 inhibitor would induce a large number of T_H_9 cells in the context of adoptive transfer treatment of tumors. To test this, we differentiated naive OT-II CD4^+^ T cells into T_H_9 cells with Jo2 stimulation or Jo2 stimulation plus SB203580 treatment (FasL + p38i-T_H_9). In a B16F10-OVA tumor model established by subcutaneous inoculation, we found that FasL + p38i-T_H_9 showed stronger antitumor effects than FasL-T_H_9 (Fig. 7h, i). To elucidate the role of IL-9 in the antitumor effects of these cells, we subcutaneously challenged *Il9r*^−*/−*^ mice with B16F10-OVA tumor cells and found that neither FasL + p38i-T_H_9 nor FasL-T_H_9 could inhibit tumor progression (Fig. 7j, k). These results indicated that FasL + p38i-T_H_9 are promising for the adoptive transfer treatment of tumors.

### Fas-related T_H_9 cells indicate a better prognosis

**Fig. 8:**
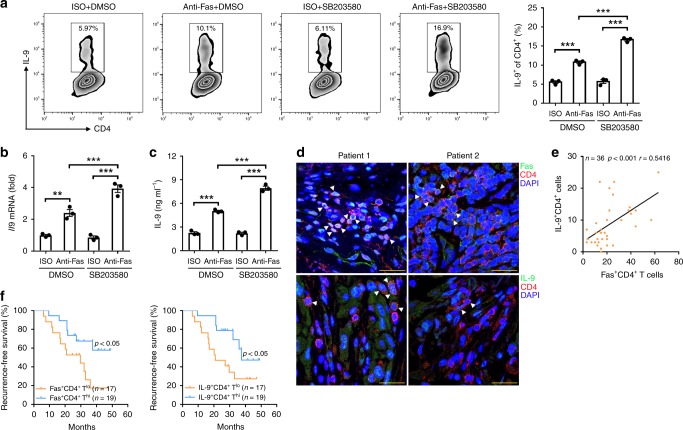
Fas-related T helper type 9 (T_H_9) cells indicate a good prognosis. **a**–**c** Flow cytometric analysis of the frequency of IL-9^+^ (interleukin-9^+^) cells among CD4^+^ T cells (left) and the corresponding statistical analysis (right) (**a**), real-time PCR analysis of *Il9* messenger RNA (mRNA) expression in T_H_9 cells (**b**), and enzyme-linked immunosorbent assay (ELISA) measurement of the IL-9 level (**c**) in T_H_9 cells after the stimulation of human naive CD4^+^CD45RA^+^CD45RO^−^ T cells with 10 μg ml^−1^ ISO or antibodies against human Fas (anti-Fas) with or without the p38 inhibitor SB203580 (0.4 μM) under T_H_9-skewing conditions for 4 days (*n* = 3). **d** Representative immunofluorescence staining of Fas^+^CD4^+^ and IL-9^+^CD4^+^ T cells in tumor tissues from non-small-cell lung carcinoma (NSCLC) patients. Arrows indicate Fas^+^CD4^+^ or IL-9^+^CD4^+^ T cells. **e** Pearson’s correlation between Fas^+^CD4^+^ T cells and IL-9^+^CD4^+^ T cells in tumor tissues from NSCLC patients. **f** The relationship between the recurrence-free survival rate of NSCLC patients and the corresponding Fas^+^CD4^+^ or IL-9^+^CD4^+^ T cell numbers in tumor tissues. Scale bar = 25 μm. **P < 0.01 and ***P < 0.001 (unpaired Student’s *t* test: **a** (right)–**c**, Spearman’s rank-order correlation test: **e**; or log-rank test: **f**). Representative results from three independent experiments are shown (mean and s.d.)

To extend our findings to humans, we investigated the effect of Fas ligation on human IL-9-producing T cell induction and found that Fas ligation significantly improved human IL-9-producing T cell generation (Fig. 8a–c). When Fas ligation was combined with a p38 inhibitor, this tendency was more evident (Fig. 8a–c). Then, we detected Fas and IL-9 expression in CD4^+^ T cells from tumor tissues in a cohort of 36 cancer patients with non-small-cell lung carcinoma (NSCLC) (Supplementary Table 1). Immunofluorescence staining revealed that the patients with high numbers of Fas^+^CD4^+^ T cells often exhibited high numbers of IL-9^+^CD4^+^ T cells (Fig. 8d). Further analysis confirmed the positive correlation between the numbers of Fas^+^CD4^+^ T cells and IL-9^+^CD4^+^ T cells in the tumor tissues (Fig. 8e). To confirm that T_H_9 cells also have antitumor activity in humans, we divided the patients into high Fas^+^CD4^+^ T cell number (Fas^+^CD4^+^ T^hi^) and low Fas^+^CD4^+^ T cell number (Fas^+^CD4^+^ T^lo^) groups or high IL-9^+^CD4^+^ T cell number (IL-9^+^CD4^+^ T^hi^) and low IL-9^+^CD4^+^ T cell number (IL-9^+^CD4^+^ T^lo^) groups and found that the recurrence-free survival in both the Fas^+^CD4^+^ T^hi^ and IL-9^+^CD4^+^ T^hi^ patient groups was better than that in the Fas^+^CD4^+^ T^lo^ and IL-9^+^CD4^+^ T^lo^ patient groups, respectively (Fig. 8f). Collectively, these results demonstrated that Fas signaling also promotes human IL-9-producing T cell induction, which has a beneficial effect on the outcomes of patients with tumor.

## Discussion

Fas signaling is important in the induction of apoptosis in activated T cells. However, there were no obvious differences in apoptosis or proliferation between WT-T_H_9 and *Fas*^*lpr*^-T_H_9 after transfer into IBD mice. This lack of difference might have been caused by the short course of acute IBD, or the Fas levels in the WT-T_H_9 might not have been sufficient to trigger apoptotic signaling when we evaluated them. However, Fas-mediated non-apoptotic signaling was observable because the mice receiving the transfer of WT CD4^+^ T cells differentiated under T_H_9-skewing conditions showed IBD symptoms with increased severity dependent on IL-9. Therefore, non-apoptotic Fas signaling is critical to generate IL-9-producing T cells and induce IL-9-mediated physiological functions.

Under T_H_9-skewing conditions with Fas ligation, TCR signaling activates PKCθ, mediating NF-κB activation. We demonstrated that Fas signaling activated PKCβ, which was necessary for increased NF-κB-dependent IL-9-producing cell induction. PKCθ was not implicated in Fas ligation-mediated IL-9-producing T cell induction because enzastaurin completely abolished this induction. Additionally, activated PKCβ could activate p38, which subsequently phosphorylated NFAT1, leading to NFAT1 inactivation. Since NFAT1 cooperates with NF-κB to promote T_H_9 cell differentiation, Fas ligation initiates both positive and negative signaling for T_H_9 cell differentiation. However, Fas signaling still markedly promoted T_H_9 cell differentiation, indicating the decisive role of NF-κB.

p38 inhibitors have been demonstrated to exert antitumor effects^58,59^, but serious side effects resulting from the systemic administration of SB203580 restrain its application. Here, we showed that systemic administration of low-dose SB203580 did not cause obvious side effects. Interestingly, we elucidated that even at very low doses, SB203580 could effectively initiate a T_H_9 cell-dependent antitumor effect. Moreover, T_H_9 cells induced by Fas plus SB203580 showed relatively strong antitumor efficacy. Given the important role of T_H_9 cells in antitumor immunity, SB203580 treatment is potential strategy for cancer therapy.

After their localizing to the plasma membrane, classic PKCs can be activated by calcium^40^. Both Fas and TCR signaling can initiate Ca^2+^ flux. It is difficult to discriminate between Ca^2+^ flux induced by Fas and that induced by TCR signaling. Fas signaling participated in PLCγ activation and the subsequent increase in Ca^2+^ flux. Fas also interacted with Zap-70, mediating PLCγ activation. The Fas mutations Y224A and Y274A abolished the interaction between Fas and Zap-70 and T_H_9 cell differentiation induced by Fas ligation. Therefore, Ca^2+^ flux induced by Fas signaling is indispensable for PKCβ activation and subsequent T_H_9 cell differentiation. The role of the Ca^2+^ flux induced by TCR signaling in the activation of PKCβ needs further study. Interestingly, Tyr284 is located in the YXXL motif of Fas, which is necessary for the binding between Fas and Zap-70^47^. However, according to our results, the Y284A mutation in Fas had no effect on the interaction betwen Fas and Zap-70 or on the Fas ligation-induced induction of IL-9-producing T cells. This finding shows that Tyr224 and Tyr274, but not Tyr284, are responsible for Fas ligation-induced IL-9-producing T cell generation. Thus, we revealed novel functional Tyr sites in Fas.

Fas has been reported to promote T_H_17 cell development, but inhibit T_H_1 cell development by sequestering STAT1^24^. We also detected decreased T_H_17 cell differentiation and increased T_H_1 cell differentiation in CD4^+^ T cells with Fas deficiency in vitro. However, in cells with a Fas defect, we detected reduced T_H_9 cell generation, but the generation of T_H_17 and T_H_1 cells remained unchanged in tumor-bearing mice. This result may be attributable to the different mice or different disease models we used. The same TF can play roles in the differentiation of different T_H_ cell subsets. STAT1 is also involved in reinforcing T_H_9 cell development^12^. However, Fas signaling-mediated T_H_9 cell differentiation is STAT1 independent. Therefore, the different environments that CD4^+^ T cells encounter determine their differentiation fates. T_H_9-skewing conditions are probably dominant in tumor-bearing mice.

Previous publications demonstrated IL-9- and IL-21-dependent antitumor effects of T_H_9 cells^12,55^. In these papers, the authors also verified that T_H_9 cells inhibit tumor growth by activating CD8^+^ T cells^12,55^. In another publication, the authors demonstrated that T_H_9 cells are directly cytotoxic to tumor cells and that the antitumor effects of T_H_9 cells are mast cell dependent^11^. A recent report showed that IL-9 and CD8^+^ T cells only slightly affect the antitumor efficacy of T_H_9 cells, while the *Eomes*-dependent granzyme-mediated cytolytic activity and TRAF6-driven hyperproliferation of T_H_9 cells are responsible for their antitumor efficacy^13^. Our data suggested that Fas signaling promoted IFN-γ production in CD8^+^ T cells. Fas signaling also upregulated granzyme expression, which appeared to be *Eomes* independent because a Fas defect induced rather than inhibited *Emos* expression in T_H_9 cells. Overall, the Fas signaling-mediated antitumor efficacy of T_H_9 cells is IL-9 dependent. Although the antitumor effect of T_H_9 cells has been well established in mice^11–13^, the antitumor effect of T_H_9 cells on humans is not well defined. Our results demonstrated that Fas ligation promoted human T_H_9 cell differentiation. The number of Fas^+^CD4^+^ T cells and IL-9^+^CD4^+^ T cells in tumor tissues was positively correlated, and higher T_H_9 cell numbers in patients with tumor indicated a better prognosis than lower T_H_9 cell numbers. Therefore, our findings provide evidence that T_H_9 cells also have antitumor effects on humans.

In summary, we demonstrate that Fas signaling promotes T_H_9 cell differentiation through PKC-β-mediated activation of the NF-κB pathway. At the same time, PKC-β-activated p38 inactivates NFAT1 and abolishes the cooperative effect of NFAT1 on NF-κB, providing negative feedback to Fas-induced T_H_9 cell differentiation (Supplementary Fig. 8).

## Methods

### Human samples

Human blood from healthy volunteers and human lung cancer tissue samples were obtained from Zhejiang Cancer Hospital. The collection of human samples was approved by the local Ethical Committee and the Review Board of Zhejiang Cancer Hospital. All the patients were informed of the usage of their tissue samples, and signed consent forms were obtained.

### Mice and cell lines

Female C57BL/6J (6- to 8-week-old) mice and BALB/C-nu/nu mice were purchased from Joint Ventures Sipper BK Experimental Animal Co (Shanghai, China). *Fas*^*lpr*^ and *Fasl*^*gld*^ mice were purchased from the Jackson Laboratory (Farmington, CT, USA). *Il9r*^*−/−*^^60^ mice were kindly provided by Dr. Jean-Christophe Renauld (Université Catholique de Louvain, France). Mice were housed in a specific pathogen-free facility, and experimental protocols were approved by the Animal Care and Use Committee of the School of Medicine, Zhejiang University. Murine B16F10 tumor cells and HEK293 cells were obtained from the American Type Culture Collection (Manassas, VA, USA). B16F10-OVA and LLC-OVA cells were provided by Dr. Qibin Leng (University of Chinese Academy of Sciences, Shanghai, China) and Wei Yang (Southern Medical University, Guangzhou, Guangdong, China), respectively. B16F10 and B16F10-OVA cells were cultured in Dulbecco’s modified Eagle’s medium (DMEM) supplemented with 10% (v/v) fetal calf serum (Lonza, Basel, Switzerland). LLC-OVA and HEK293 cells were cultured in RPMI-1640 medium supplemented with 10% fetal calf serum. All cells were routinely tested for mycoplasma contamination using the Mycoplasma Detection Kit (Lonza) and were found to be negative.

### In vitro differentiation of T cells

Naive CD4^+^CD62L^hi^CD44^lo^ T cells were obtained from the spleen and lymph nodes of mice. Sorted naive CD4^+^ T cells were routinely 98% pure. The sorted naive CD4^+^ T cells were stimulated with plate-bound anti-CD3 (145-2C11, 2 μg ml^−1^, Bio X cell, West Lebanon, NH, USA) and anti-CD28 (PV-1, 2 μg ml^−1^, Bio X cell) antibodies, and polarized into effector CD4^+^ T lymphocyte subsets without cytokines, and with anti-IFN-γ (BE0054, 10 μg ml^−1^, Bio X cell) and anti-IL-4 (BE0045, 10 μg ml^−1^, Bio X cell) (T_H_0 cells); with IL-12 (130-096-707, 20 ng ml^−1^, Miltenyi Biotec, Bergisch Gladbach, Germany) and anti-IL-4 (10 μg ml^−1^) for T_H_1 cells; with IL-4 (130-094-061, 20 ng ml^−1^, Miltenyi Biotec) and anti-IFN-γ (10 μg ml^−1^) for T_H_2 cells; with TGF-β1 (130-095-067, 2 ng ml^−1^, Miltenyi Biotec), IL-4 (20 ng ml^−1^), and anti-IFN-γ (10 μg ml^−1^) for T_H_9 cells; with TGF-β1 (2 ng ml^−1^), IL-6 (130-094-065, 25 ng ml^−1^, Miltenyi), anti-IFN-γ (10 μg ml^−1^), and anti-IL-4 (10 μg ml^−1^) for T_H_17 cells; or with TGF-β1 (2 ng ml^−1^), anti-IFN-γ (10 μg ml^−1^), and anti-IL-4 (10 μg ml^−1^) for T_reg_ cells. In some experiments, antibodies against murine Fas (Jo2, 10 μg ml^−1^, Thermo Fisher Scientific, Waltham, MA, USA), z-VAD-fmk (S8102, 1 μM, Selleck, Houston, TX, USA), BAY 11-7082 (S2913, 0.4 μM, Selleck), LY2409881 (S7697, 0.5 μM, Selleck), enzastaurin (S1055, 0.5 μM, Selleck), Go 6983 (S2911, 0.01 μM, Selleck), 2-APB (ab120124, 10 μM, Abcam, Cambridge, MA, USA), XC (ab120914, 10 μM, Abcam), U73122 (S8011, 0.1 μM, Selleck), manoalide (ab141554, 10 μM, Abcam), SB203580 (S1076, 0.4 μM, Selleck), FK506 (S5003, 5 pM, Selleck), or INCA-6 (sc-203160, 50 nM, Santa Cruz, Santa Cruz, CA, USA) was added at the beginning of culture. Cells were classically harvested on day 3 for the detection of cytokines by enzyme-linked immunosorbent assay (ELISA) and real-time PCR analyses. For human in vitro CD4^+^ T cell differentiation, naive CD4^+^CD45RA^+^CD45RO^−^ T cells were isolated from the peripheral blood mononuclear cells of healthy donors with the Human Naive CD4^+^ T Cell Isolation Kit II (STEMCELL, Vancouver, BC, V6A 1B6, Canada), stimulated with plate-bound anti-CD3 (5 μg ml^−1^, Bio X cell) and anti-CD28 (5 μg ml^−1^, BioLegend, San Diego, CA, USA) antibodies, and polarized into T_H_9 cells with human TGF-β1 (10 ng ml^−1^) and IL-4 (5 ng ml^−1^) (R&D Systems, Minneapolis, MN, USA).

### Immunoblotting analysis

Purified naive CD4^+^ T cells were differentiated into T_H_9 cells for different times. Then, the cells were pelleted by centrifugation for 5 min at 2000 × *g* and lysed for 30 min at 4 °C in RIPA buffer (50 mM Tris-HCl (pH 7.5), 150 mM NaCl, 1% NP-40, 0.5% sodium deoxycholate, 0.1% sodium dodecyl sulfate, 1 mM EDTA, and protease inhibitors). Subsequently, the cell lysates were separated by sodium dodecyl sulfate-polyacrylamide gel electrophoresis (10–12%) and transferred onto a polyvinylidene difluoride membrane (Millipore, Billerica, MA, USA). The membrane was blocked with 5% bovine serum albumin (BSA) in TBST buffer and then incubated with primary antibodies overnight at 4 °C. After incubating with the corresponding horse radish peroxidase-conjugated secondary antibodies for 1 h, the chemical signal were developed by NcmECL Ultra (P10100A, P10100B, New Cell & Molecular Biotech Co. Ltd, Suzhou, Jiangsu, China), and then the membrane was scanned using the Tanon 4500 Gel Imaging System. Uncropped blotting scans were presented in the affiliated Source Data file. The antibodies were diluted with NCM universal antibody diluent (WB500D, New Cell & Molecular Biotech Co. Ltd.). The antibodies used and the corresponding dilutions are listed in Supplementary Table 2.

### Ca^2+^ flux

Sorted naive CD4^+^ T cells were labeled with 4 μg ml^−1^ Fluo4 (Invitrogen, New York, NY, USA) for 1 h at 37 °C, washed with ice-cold phosphate-buffered saline (PBS), and resuspended in PBS. The labeled cells were stimulated with ISO, Jo2, U73211, or Manoalide and immediately subjected to flow cytometry analysis. Mean fluorescence ratios were plotted after analysis with FlowJo software (TreeStar, Ashland, OR, USA).

### Transfection of siRNA

Transient small interfering RNA (siRNA) transfection into naive CD4^+^ T cells was performed in vitro using TransIT-TKO (Mirus Bio, Madison, WI, USA) according to the manufacturer’s instructions. Twenty-four hours after transfection, the CD4^+^ T cells were stimulated with plate-bound anti-CD3 and anti-CD28 antibodies, differentiated into T_H_9 cells as described above, and cultured for an additional 72 h after analysis with an siRNA specific for murine *Fas*, *Zap70* (sc-29312 or sc-36867, respectively, Santa Cruz), or *p38α* (#6417, Cell Signaling), or NC siRNA (sc-37007, Santa Cruz).

### Real-time PCR

Total RNA was extracted from T cells using TRIzol reagent (Thermo Fisher Scientific) following the manufacturer’s instructions. Complementary DNAs (cDNAs) was synthesized using a cDNA Synthesis Kit (TaKaRa, Kusatsu, Shiga, Japan) following the manufacturer’s instructions. Real-time PCR was conducted using SYBR Green (TaKaRa). The following PCR conditions were used: 1 cycle at 95 °C for 30 s, 40 cycles of 95 °C for 5 s, and 60 °C for 34 s. Real-time PCR was performed with an Applied Biosystems 7500 real-time PCR system. The primers used are listed in Supplementary Table 3.

### Measurement of cytokine levels

After 72 h of polarization, cell culture supernatants were assayed by ELISA to measure the levels of mouse IFN-γ, IL-4, IL-9, and IL-17A (BioLegend) according to the manufacturer’s instructions. For intracellular staining, cells were cultured for 3 days, restimulated for 1 additional day, and then stimulated for 4 h at 37 °C in RPMI-1640 medium containing phorbol 12-myristate 13-acetate (50 ng ml^−1^, Sigma-Aldrich, St Louis, MO, USA) and ionomycin (1 μg ml^−1^, Sigma-Aldrich). After staining for surface markers, the cells were fixed and permeabilized according to the manufacturer’s instructions (Cytofix/Cytoperm Kit, Thermo Fisher Scientific) and then stained for intracellular products. The following monoclonal antibodies were used for the flow cytometric analyses: fixable viability dye eFluor^TM^ 450- or phycoerythrin-conjugated anti-CD4 and allophycocyanin-conjugated anti-IFN-γ, anti-IL-4, anti-IL-9, anti-IL-17A, or anti-Foxp3. All events were acquired on a BeckmanCoulter DxFLEX flow cytometer equipped with CytExpert experiment-based software (BeckmanCoulter, Inc.), and data were analyzed using FlowJo software (TreeStar). Gating strategies were presented in Supplementary Fig. 9.

### Retroviral infection of CD4^+^ T cells

Retroviruses were produced by transfecting Plat-E cells with 7.5 μg of pMX-IRES-GFP, pMX-Fas-WT IRES-GFP, pMX-Fas-T189A IRES-GFP, pMX-Fas-T224A IRES-GFP, pMX-Fas-T274A IRES-GFP, or pMX-Fas-T283A IRES-GFP. Cell culture medium was replaced with fresh medium after 10 h, and the retrovirus-containing supernatant was collected after an additional 72 h. To infect T cells, naive CD4^+^ T cells were first stimulated with anti-CD3 and anti-CD28 antibodies. At 24- and 36-h time points, the activated T cells were infected for 1 h by centrifugation at 1500 × *g* with 500 μl of viral supernatant in the presence of 10 μg ml^−1^ polybrene and incubated at 37 °C for an additional 1 h before the cells were removed from the viral supernatant and resuspended in the indicated T cell differentiation medium for 4 days.

### Immunofluorescence and confocal microscopy

ISO- or Jo2-treated CD4^+^ T cells incubated with or without 2-APB or XC for 15 min were fixed in prewarmed 4% paraformaldehyde for 30 min and permeabilized with 0.1% Triton X-100 for 10 min. After blocking with 5% BSA, the cells were incubated at 4 °C overnight with anti-Fas (4C3, Cell Signaling) and anti-PKCβ1 (A10-F, Abcam) or anti-PKCβ2 (EPR18104, Abcam) antibodies. Primary antibodies were detected using DyLight 488- and DyLight 549-labeled secondary antibodies (Abcam). Nuclei were stained with 4′,6-diamidino-2-phenylindole (Invitrogen). Human paraffin-embedded lung tumor sections were subjected to immunofluorescence staining. The stained tissue sections were viewed under an Olympus FluoView FV1000 confocal microscope and imaged using an Olympus FluoView version 1.4a viewer (Olympus Corp, Tokyo, Japan). Images of the cells and sections were captured, and positive areas were analyzed.

### RNA-seq analysis

Total RNA was isolated and reverse transcribed into cDNA to generate an indexed Illumina library, followed by sequencing at the Beijing Genomics Institute (Beijing, China) using a BGISEQ-500 platform. High-quality reads were aligned to a mouse reference genome (GRCm38) by Bowtie2. The expression levels of individual genes were normalized to the FKPM (fragments per kilobase million) reads by RNA-seq by an expectation maximization algorithm. Significant differential expression of a gene was defined as a >2-fold expression difference vs. the control with an adjusted *P* value <0.05. A heat map was analyzed by Gene Ontology using Cluster software and visualized with Java Treeview. DEGs were analyzed by Gene Ontology using the AMIGO and DAVID software. The enrichment degrees of DEGs were analyzed using Kyoto Encyclopedia of Genes and Genomes annotations.

### Cell viability assay

LLC-OVA cell viability was measured using a CCK-8 assay according to the manufacturer’s instructions. LLC-OVA cells were stimulated with dimethyl sulfoxide (DMSO) or 0.15, 10, 20, or 30 μg ml^−1^ SB203580 for 72 h in a 96-well plate, and 10 μl of CCK-8 (MultiSciences, Hangzhou, Zhejiang, China) was added per well and incubated for 2 h at 37 °C. A multiplate reader was used to measure the absorbance at 450 nm. Cell viability was expressed as the absorbance at 450 nm.

### Chromatographic conditions and data acquisition

Mice received an intraperitoneal injection of DMSO or 0.5 or 10 mg kg^−1^ SB203580. After 0.5, 1, 3, or 6 h, plasma was collected and centrifuged at 12,000 × *g* for 10 min, and the supernatants were subjected to chromatographic detection. High-performance liquid chromatography analyses were carried out using Agilent 1100 series LC equipment. The elution system was set up as follows: an Inertil ODS-4 column (4.6 × 250 mm^2^, 5 μm) was eluted using a water and methanol gradient at 40 °C with a constant flow rate of 1.0 ml min^−1^ (0–20 min, 50–100% methanol; 20–25 min, 100% methanol). The fluorescence detector was set at 325 nm (excitation wavelength) and 408 nm (emission wavelength). Under these chromatographic conditions, the retention time of SB203580 was 13.0 min. The acquired data were processed using the ChemStation for LC 3D software. To obtain chromatograms, data were extracted from the software and plotted in the Microsoft Excel 2010 program.

### Transfer of T_H_9 cells into IBD mice

WT and *Il9r*^*−/−*^ mice were randomized to produce groups with similar average body weights. Acute IBD was induced by administering 2.5% (w/v) DSS (MP Biomedicals, Solon, OH, USA) with a molecular weight of 36,000–50,000 in acidified drinking water for 11 days. The day that the mice started to drink the DSS solution was defined as day 0. For in vivo T_H_9 cell transfer, WT mice were intravenously injected with 2 × 10^6^ cT_H_9, FasL-T_H_9, WT-T_H_9, or *Fas*^*lpr*^-T_H_9 cells on day 0. For in vivo blockade of IL-9 function, WT mice were intraperitoneally injected with 100 μg of anti-IL-9 (MM9C1, Bio X Cell) every other day. Mouse IgG2a (BE0085, Bio X Cell) was used as the isotype control antibodies.

### Colon tissue culture and isolation of LPLs

Colons were incised longitudinally and washed four times in Hank’s buffered salt solution supplemented with penicillin and streptomycin. One-centimeter-long transverse segments were prepared and cultured in serum-free RPMI-1640 medium supplemented with penicillin, streptomycin, l-glutamine, and nonessential amino acids. After 24 h, the supernatants were collected, and the production of IL-6, TNF, IL-1β, IL-10, and IL-22 was measured using ELISAs (BioLegend).

For the isolation of LPLs, small intestines were cut open longitudinally after removing Peyer’s patches and washed with DMEM. Then, the open small intestines were cut into pieces approximately 5 mm in length, and these pieces were incubated in prewarmed DMEM containing 3% fetal calf serum, 0.2% Hank’s solution, 5 mM EDTA, and 0.145 mg ml^−1^ dithiothreitol for 10 min with constant agitation. Then, the small intestine samples were incubated in a solution of 3% DMEM, 0.2% fetal calf serum, 0.025% Hank’s solution, 50 mg ml^−1^ DNase, and 75 mg ml^−1^ collagenase II for 5 min, and then the dissociated cells were collected. Finally, the solution containing the digested tissue was passed through a 100-μm cell strainer, and LPLs were isolated on an 80%/40% Percoll (GE Healthcare, Uppsala, Sweden) gradient. The sorted LPLs were applied for the following experiment after washing with PBS.

### Tumor growth experiments

Bone marrow cells were isolated from *Fas*^*lpr*^ or WT mice, and 1 × 10^7^ cells were injected intravenously into C57BL/6J mice that had received sublethal irradiation (400 rad) 1 day before. The chimeric mice were used for tumor inoculation after 6–8 weeks. A total of 1 × 10^6^ LLC-OVA cells were injected subcutaneously into the chimeric mice or WT mice. In vivo IL-9 neutralization was achieved by intraperitoneal injection of 100 μg of anti-IL-9-neutralizing antibodies (MM9C1, Bio X Cell) every other day following tumor implantation. To evaluate the safety of systematic treatment with low- or high-dose SB203580, mice received intraperitoneal injection of SB203580 (0.5 or 10 mg kg^−1^) every other day. Body weight was monitored, and the heart, liver, spleen, lungs, and kidneys were collected for histopathological analysis 20 days later. For p38 inhibition in vivo, mice received an intraperitoneal injection of SB203580 (0.5 mg kg^−1^) every other day following tumor implantation. Tumor size was monitored every other day by Vernier calipers. According to the criteria of the Animal Care and Use Committee of the School of Medicine, Zhejiang University, when the tumor size was over 8000 mm^3^, the tumor-bearing mice were euthanized by an intraperitoneal injection of 50 mg kg^−1^ pentobarbital sodium. TILs were prepared by enzymatic digestion with 1 mg ml^−1^ collagenase (Sigma-Aldrich), 0.5 mg ml^−1^ DNase I, and 25 μg ml^−1^ hyaluronidase (Sigma-Aldrich) at 37 °C for 30 min, followed by Percoll (GE Healthcare) gradient purification. The isolated TILs were restimulated with the OVA_323–339_ or OVA_257–264_ peptides at a final concentration of 10 or 20 µg ml^−1^, respectively, in vitro.

To assess the antitumor effects of FasL-T_H_9 cells, 5 × 10^5^ B16-OVA or LLC-OVA cells were injected intravenously into C57BL/6 mice on day 0. Then, on days 1 and 6, the mice received an intravenous injection of 2 × 10^6^ effector OT-II T_H_9 cells differentiated with or without Jo2. Alternatively, 1 × 10^6^ B16-OVA cells were injected subcutaneously into C57BL/6 mice on day 0. Then, on days 1 and 6, the mice received an intravenous injection of 2 × 10^6^ effector OT-II T_H_9 cells differentiated with or without Jo2 or Jo2 combined with SB203580. Lung tumor foci were enumerated after 16 days in a blinded manner. To assess the role of IL-9 in the antitumor effects of FasL-T_H_9, 5 × 10^5^ or 1 × 10^6^ B16-OVA cells were injected intravenously or subcutaneously into *Il9r*^−^^*/−*^ mice. Then, on days 1 and 6, the mice received an intravenous injection of 2 × 10^6^ effector OT-II T_H_9 cells differentiated with or without Jo2. The tumor-bearing mice with lung metastasis were euthanized by an intraperitoneal injection of 50 mg kg^−1^ pentobarbital sodium before the onset of delayed action.

### Histopathology

Heart, liver, spleen, lungs, kidneys, and intestine were dissected from individual mice in groups and immediately fixed in 10% paraformaldehyde. The heart, liver, spleen, lung, kidney, and intestine samples were subjected to hematoxylin–eosin staining.

### Statistical analyses

Data are expressed as the mean ± standard deviation. An unpaired Student’s *t* test was used for comparisons between two groups, the log-rank test was used for survival rate analysis, and the Spearman’s rank-order correlation test was used for Pearson’s correlation analysis using the GraphPad Prism 7.0 software. A difference was considered statistically significant if the *P* value was <0.05.

### Reporting summary

Further information on research design is available in the Nature Research Reporting Summary linked to this article.

## Supplementary information

Supplementary Information

Peer Review File

Reporting Summary

Description of Additional Supplementary Files

Supplementary Data 1

Source Data

## Data Availability

The data underlying all findings of this study are available from the corresponding author upon reasonable request and are provided as a separate Source Data file. The high-throughput RNA-sequencing data have been deposited in the NCBI Sequence Read Archive under the BioProject accession number SRP159792.
