# Supplementary material for: Fas signaling-mediated TH9 cell differentiation favors bowel inflammation and antitumor functions
Source: Nat Commun. 2019 Jul 2;10:2924. doi: 10.1038/s41467-019-10889-4 (PMC6606754; doi:10.1038/s41467-019-10889-4)
Supplement: Supplementary file 1 — Supplementary Information [file 41467_2019_10889_MOESM1_ESM.pdf]

Supplementary information

Fas signaling-mediated T_H9 cell differentiation favors bowel inflammation and antitumor functions

Shen et al.

Supplementary figures 1 to 9

Supplementary tables 1 to 3

Supplementary Figure 1

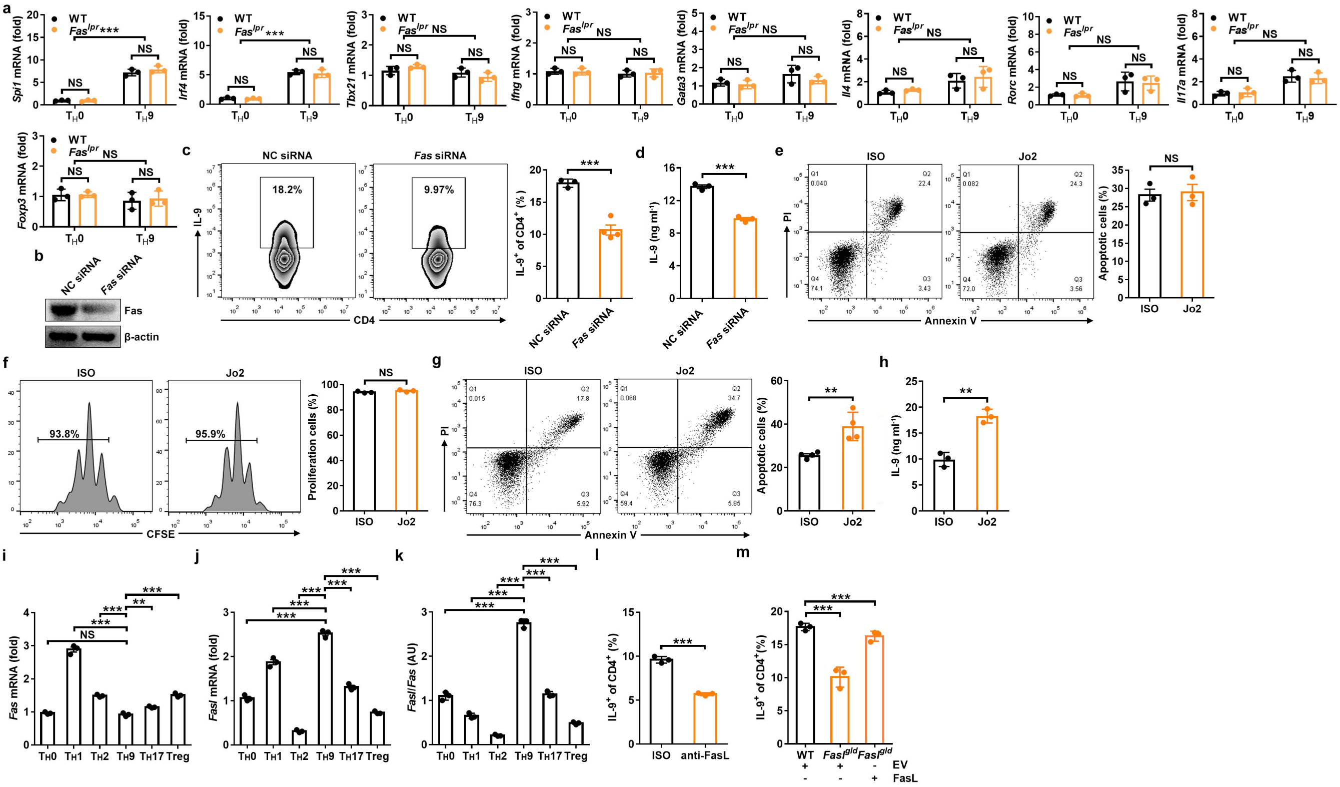

Supplementary Figure 1: Fas signaling promotes T_H9 cell differentiation *in vitro*. (**a**) Naïve $CD4^+CD62L^{hi}CD44^{lo}$ T cells were sorted from WT and *Fas*^{lpr} mice and differentiated into T_H0 and T_H9 cells for 3 days. Real-time PCR analysis of the expression of the indicated genes in T_H0 and T_H9 cells. (**b-d**) Naïve $CD4^+$ T cells from WT mice transfected with NC siRNA or *Fas* siRNA for 24 h, western blotting evaluation of the Fas protein levels (**b**), or were differentiated into T_H9 cells for another 4 days. Flow cytometric analysis of the frequency of IL-9^+ cells among the $CD4^+$ T cells (left) and the corresponding statistical analysis (right) (**c**) and ELISA measurement of IL-9 cytokines in supernatants from T_H9 cells (**d**). (**e-h**) Naïve T cells from WT mice with or without CFSE labeling were differentiated into T_H9 cells under T_H9-skewing conditions for 3 days. Flow cytometric analysis of apoptotic cells (left) and the corresponding statistical analysis (right) (**e**), flow cytometric analysis of proliferative T_H9 cells according to CFSE dilution (left) and the corresponding statistical analysis (right) (**f**), flow cytometric analysis of apoptotic cells after the restimulation of differentiated T_H9 cells with plate-bound anti-CD3 and anti-CD28 antibodies for another 24 h (left) and the corresponding statistical analysis (right) (**g**), and ELISA measurement of IL-9 cytokines in supernatants from restimulated T_H9 cells (**h**). (**i-k**) Naïve WT $CD4^+$ T cells were differentiated into T_H0, T_H1, T_H2, T_H9, T_H17 cells and Tregs for 3 days. Real-time PCR analysis of *Fas* (**i**) and *Fasl* (**j**) genes in T_H9 cells, statistical analysis of the ratio of *Fas* gene levels to *Fasl* gene levels (**k**). (**l**) Flow cytometric analysis of the frequency of IL-9^+ cells among the $CD4^+$ T cells differentiated from naïve WT $CD4^+$ T cells with or without anti-FasL for 4 days. (**m**) Flow cytometric analysis of the frequency of IL-9^+ cells among the $CD4^+$ T cells differentiated from naïve $CD4^+$ T cells from WT or *Fasl*^{gld} mice with or without FasL expressing vector transfection for 4 days. NS, not significant; $*P < 0.05$, $**P < 0.01$ and $***P < 0.001$ (unpaired Student's t test: **a, c-m** or one-way ANOVA test: **a**). Representative results from three independent experiments are shown (mean and s.d.).

Supplementary Figure 2

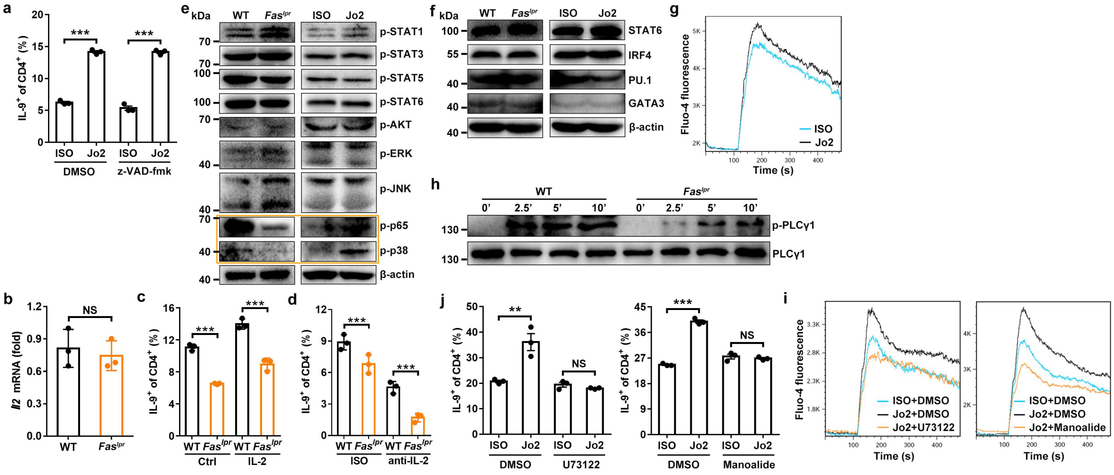

Supplementary Figure 2: Fas signaling enhances generation of IL-9-producing cells by activating Ca^{2+}-dependent NF-κB. (**a**) Flow cytometric analysis of the frequency of IL-9^{+} cells among the CD4^{+} T cells after naïve CD4^{+} T cells were stimulated with 10 μg ml^{-1} ISO or Jo2 with or without the pan-caspase inhibitor z-VAD-fmk at 1 μM, under T$_{H}$9-skewing conditions for 4 days. (**b-d**) Naïve WT and *Fas^{lpr}* CD4^{+} T cells were differentiated into T$_{H}$9 cells under T$_{H}$9-skewing conditions. Real-time PCR analysis of *Il2* gene in the cells after 24 h (**b**), flow cytometric analysis of the frequency of IL-9^{+} cells among the CD4^{+} T cells differentiated with IL-2 (**c**) or anti-IL-2 (**d**) after 4 days. (**e, f**) Western blotting analysis of the indicated phosphorylated (**e**) and TF (**f**) proteins in WT or *Fas^{lpr}* CD4^{+} T cells or WT CD4^{+} T cells stimulated with 10 μg ml^{-1} ISO or Jo2 under T$_{H}$9-skewing conditions for 15 min (**e**) or 24 h (**f**). (**g**) Flow cytometric analysis of Ca^{2+} flux in naïve CD4^{+} T cells stimulated with 10 μg ml^{-1} ISO or Jo2 under T$_{H}$9-skewing conditions over time. (**h**) Western blotting analysis of p-PLCγ1 in WT or *Fas^{lpr}* CD4^{+} T cells stimulated with 10 μg ml^{-1} Jo2 under T$_{H}$9-skewing conditions at the indicated time points. (**i, j**) Flow cytometric analysis of Ca^{2+} flux (**i**) or the frequency of IL-9^{+} cells (**j**) in the naïve CD4^{+} T cells stimulated with 10 μg ml^{-1} ISO or Jo2 with the PLC inhibitor U73122 at 0.1 μM or the irreversible PLC inhibitor manoalide at 10 μM under T$_{H}$9-skewing conditions over time (**i**) or for 4 days (**j**). NS, not significant; $**P < 0.01$ and $***P < 0.001$ (unpaired Student's *t* test). Representative results from three independent experiments are shown (mean and s.d.).

Supplementary Figure 3

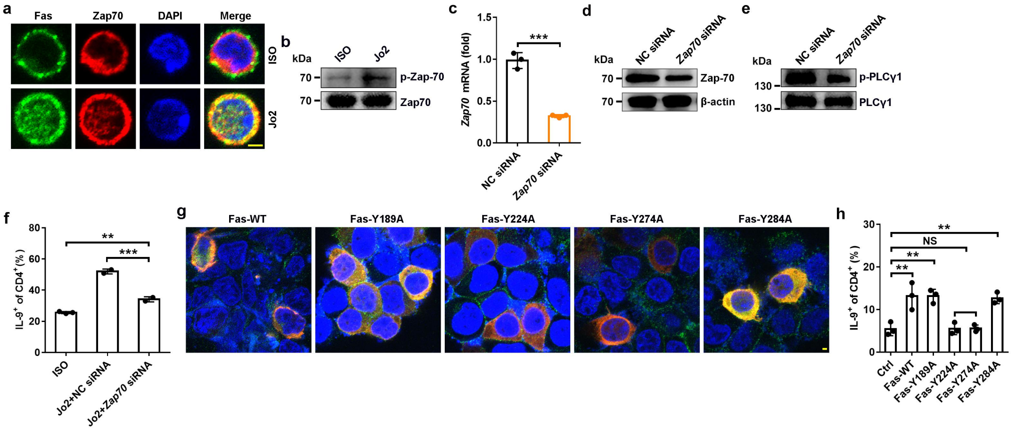

Supplementary Figure 3: Tyr224 and Tyr274 in Fas contribute to the Zap-70-dependent activation of PLCγ1. (**a, b**) Immunofluorescence staining of Fas and Zap-70 (**a**) and western blotting detection of phosphorylated Zap-70 (**b**) in naïve the $CD4^+$ T cells stimulated with 10 μg ml^{-1} ISO or Jo2 under T_H9-skewing conditions for 5 min. (**c, d**) Real-time PCR (n = 3) (**c**) or western blotting (**d**) measurement of Zap-70 gene or protein levels, respectively, in naïve the $CD4^+$ T cells transfected with *Zap70* siRNA for 24 h. (**e, f**) Western blotting detection of phosphorylated PLCγ1 (**e**) or flow cytometric analysis of the frequency of IL-9^+ cells (n = 2-3) (**f**) in naïve $CD4^+$ T cells transfected with *Zap70* siRNA followed by 10 μg ml^{-1} ISO or Jo2 stimulation under T_H9-skewing conditions for 5 min (**e**) or 4 days (**f**). (**g**) Immunofluorescence staining of Fas and Zap-70 in HEK293 cells transfected with Zap-70 and the corresponding *Fas* expression plasmids for 24 h. (**h**) Flow cytometric analysis of the frequency of IL-9^+ cells in the naïve Fas^{lpr} $CD4^+$ T infected with the indicated *Fas* expression retroviruses followed by 10 μg ml^{-1} ISO or Jo2 stimulation under T_H9-skewing conditions for 4 days (n = 3). Scale bar = 2 μm. NS, not significant; *$P < 0.05$, **$P < 0.01$ and *** $P < 0.001$ (unpaired Student's *t* test: **c, f** and **h**). Representative results from three independent experiments are shown (mean and s.d.).

Supplementary Figure 4

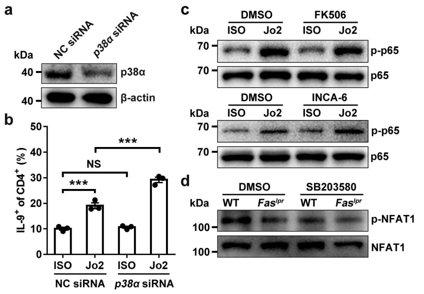

Supplementary Figure 4: p38 inhibits Fas signaling-mediated induction of IL-9-producing T cells by decreasing NFAT1 activation. (**a**) Western blotting analysis of p38α in the naïve CD4$^+$ T cells after transfection with *p38α* siRNA for 24 h. (**b**) Flow cytometric analysis of the frequency of IL-9$^+$ cells among the CD4$^+$ T cells after the naïve CD4$^+$ T cells were transfected with *p38α* siRNA for 24 h and then stimulated with 10 μg ml^{-1} ISO or Jo2 under T$_H$9-skewing conditions for 4 days. (**c**) Western blotting analysis of p-p65 proteins in the naïve CD4$^+$ T cells stimulated with 10 μg ml^{-1} ISO or Jo2 for 15 min with or without 5 pM FK506 or 50 nM INCA-6, under T$_H$9-skewing conditions. (**d**) Western blotting analysis of p-NFAT1 proteins in WT or *Fas^{lpr}* CD4$^+$ T cells with or without 0.4 μM SB203580 under T$_H$9-skewing conditions for 15 min. NS, not significant; ****P* < 0.001 (unpaired Student's *t* test: **b**). Representative results from three independent experiments are shown (mean and s.d.) (n = 3).

Supplementary Figure 5

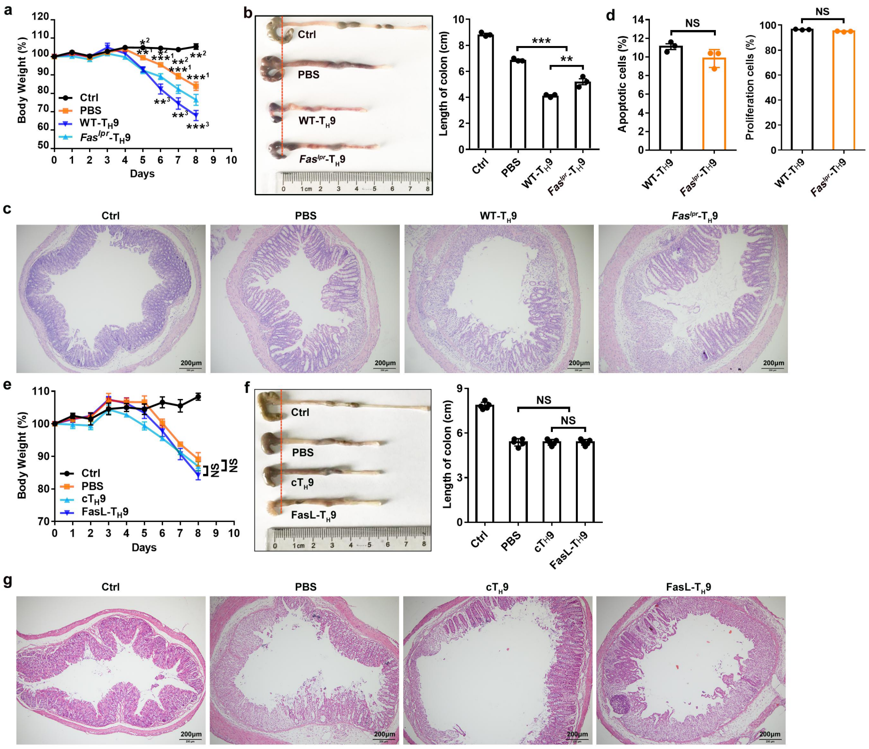

Supplementary Figure 5: FasL-T$_H$9 exacerbate murine IBD via IL-9. (**a-c**) Weight (n = 5) (**a**), colonic length (**b**) and hematoxylin-eosin staining of colonic tissue sections (**c**) of 2.5% DSS (w/v)-induced IBD mice that received an intravenous transfer of 2 × 10^6 WT-T$_H$9 or *Fas^{lpr}*-T$_H$9. (**d**) Flow cytometric detection of apoptosis and proliferation of CFSE$^+$ WT-T$_H$9 or *Fas^{lpr}*-T$_H$9 in mLNs of 2.5% DSS (w/v)-induced IBD mice that received an intravenous transfer of 1 × 10^7 CFSE-labeled WT-T$_H$9 or *Fas^{lpr}*-T$_H$9 for 5 days (n = 3). (**e-g**) Weight (n = 5) (**e**), colonic length (**f**) and hematoxylin-eosin-stained colonic tissue sections (**g**) of 2.5% DSS (w/v)-induced IBD *Il9r$^{-/-}$* mice that received an intravenous transfer of 2 × 10^6 cT$_H$9 or FasL-T$_H$9. Scale bar = 200 μm. NS, not significant; *P < 0.05, **P < 0.01 and ***P < 0.001 (unpaired Student's *t* test: **a, b** (right)**, d, e** and **f** (right). 1, compared with WT-T$_H$9; 2, compared with *Fas^{lpr}*-T$_H$9; and 3, compared with *Fas^{lpr}*-T$_H$9. Representative results from three independent experiments are shown (mean and s.d.).

Supplementary Figure 6

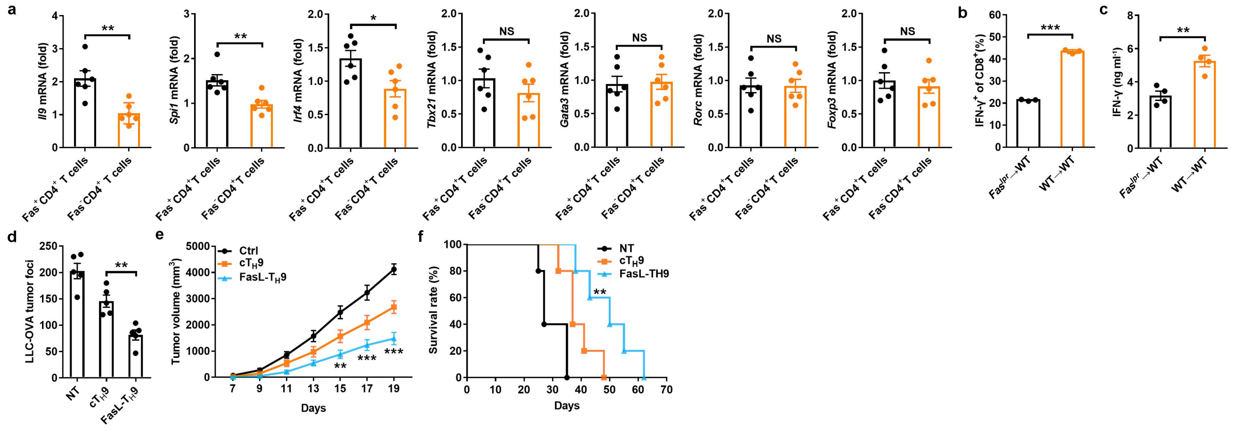

Supplementary Figure 6: Fas signaling is involved in the antitumor activity of T_H9 cells. (a) Real-time PCR analysis of the indicated genes in Fas^+CD4^+ and Fas^-CD4^+ T cells from the lung-draining lymph nodes of mice that received an intravenous injection of B16F10 melanoma cells for 16 days (n = 6). (**b**) Flow cytometric analysis of the frequency of IFN-γ^+ cells among the $CD8^+$ T cells in TILs from WT mice reconstituted with bone marrow cells from WT or *Fas^{lpr}* mice for 2 months, followed by subcutaneous injection of LLC-OVA lung tumor cells for 20 days (n = 3). (**c**) ELISA measurement of IFN-γ secreted by $OVA_{257\text{-}264}$-stimulated TILs from the mice described in **b** (n = 4). (**d**) Lung tumor foci of WT mice 16 days after an intravenous injection of LLC-OVA cells with NT or transfer of OT-II cT_H9 or FasL-T_H9 1 and 6 days later (n = 5). (**e, f**) Tumor growth (**e**) and survival (**f**) of WT mice that received a subcutaneous injection of LLC-OVA cells followed by NT or an intravenous injection of 2×10^6 OT-II cT_H9 or FasL-T_H9 1 and 6 days later (n = 5). NS, not significant; $*P < 0.05$, $**P < 0.01$ and $***P < 0.001$ (unpaired Student's *t* test: **a-e** or log-rank test: **f**). Compared with cT_H9 in **e** and **f**. Representative results from three independent experiments are shown (mean and s.d.).

Supplementary Figure 7

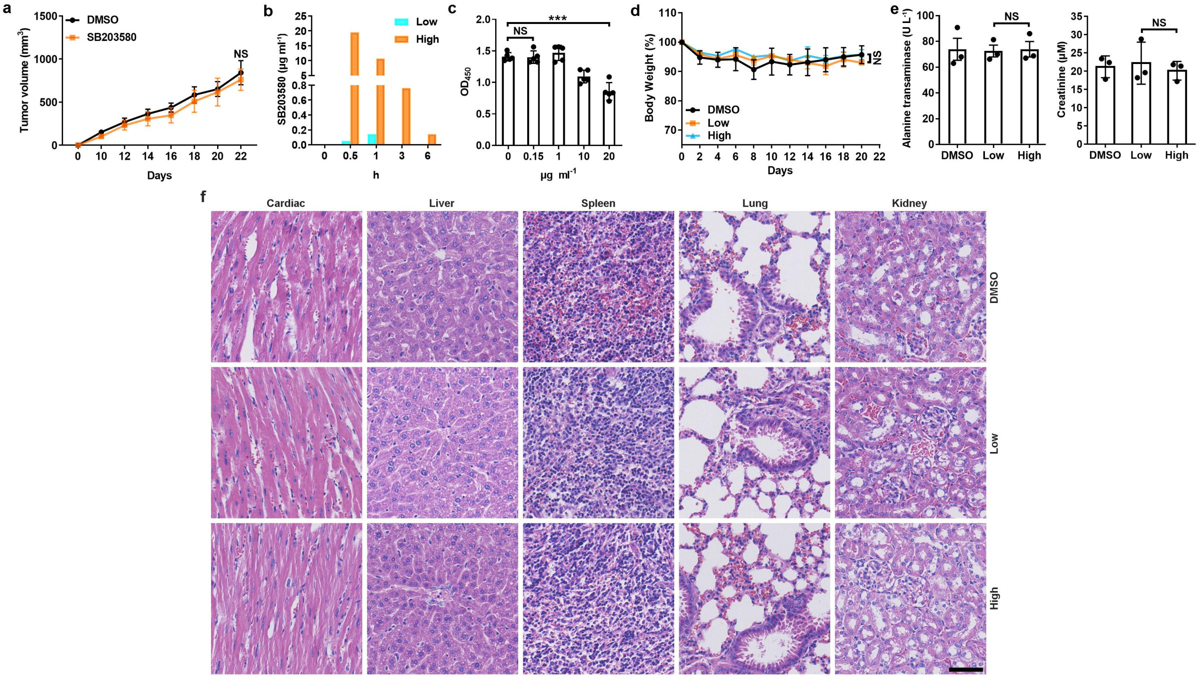

Supplementary Figure 7: Low-dose SB203580 treatment has no obvious side effects *in vivo.* (**a**) Tumor growth of nu/nu mice that received a subcutaneous injection of LLC-OVA cells followed by an intraperitoneal injection of SB203580 (0.5 mg kg^{-1}) every other day (n = 5). (**b**) Liquid chromatography monitoring of the concentration of SB203580 in murine plasma at the indicated time points after an intraperitoneal injection of low- or high-dose SB203580. (**c**) CCK-8 measurement of LLC-OVA cell viability following treatment with the indicated dose of SB203580 for 24 h (n = 3). (**d-f**) Assessment of weight (n = 3) (**d**), serum alanine transaminase and creatinine (n = 3) (**e**), and representative pictures of hematoxylin-eosin staining (**f**) of mice received an intraperitoneal treatment with low (0.5 mg kg^{-1}) or high (10 mg kg^{-1}) dose SB203580 every other day from day 0. Scale bar = 50 µm. NS, not significant; $^{***}P < 0.001$ (unpaired Student's *t* test: **a** and **c-e**). Representative results from three independent experiments are shown (mean and s.d.).

Supplementary Figure 8

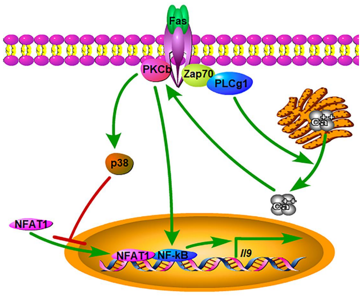

Supplementary Figure 8: Working model of Fas-signaling to control T_H9 cell differentiation. Fas signaling promotes T_H9 cell differentiation through PKC-β-mediated activation of the canonical NF-κB pathway. At the same time PKC-β-activated p38 inactivates NFAT1 and abolishes its cooperative effect on NF-κB, providing a negative feedback to Fas-induced T_H9 cell differentiation.

Supplementary Figure 9

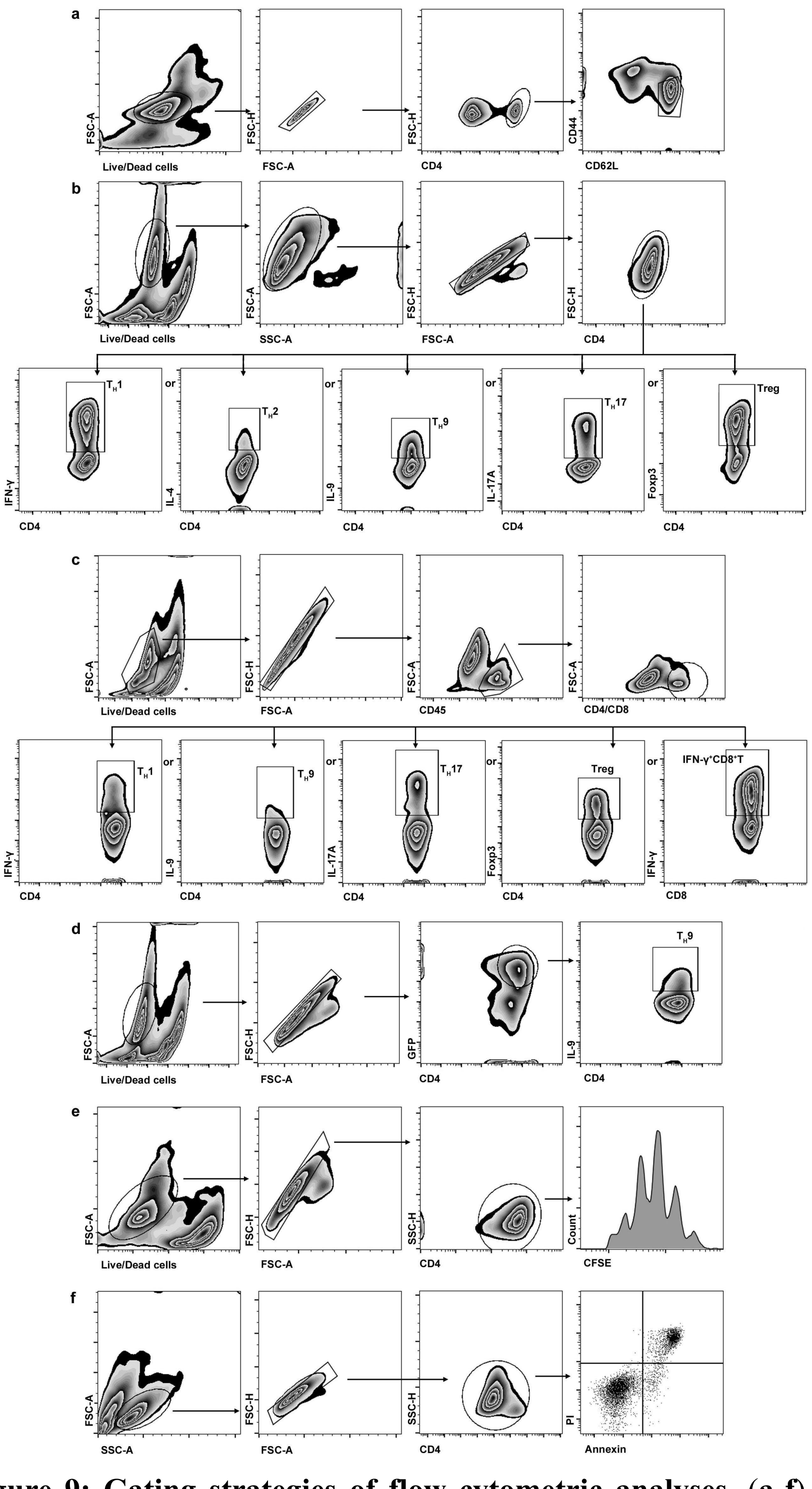

Supplementary Figure 9: Gating strategies of flow cytometric analyses. (**a-f**) Gating strategies of naïve $CD4^+CD62L^{hi}CD44^{lo}$ T cell sorting (**a**), figures 1a, 1d, 1g, 3b, 3e, 3h, 4a, 4c, 4g and 8a, and supplementary figures 1c, 1i, 2a, 2c, 2d, 2j, 3f and 4b (**b**), figures 6c and 7c, and supplementary figure 6b (**c**), supplementary figures 1m and 3h (**d**), supplementary figures 1f and 5d (**e**), supplementary figures 1e, 1g and 5d (**f**).

Supplementary Table 1: Basic information of lung cancer patients

**Characteristics**	
**Number**	36
**Age (years)**	59.39 ± 7.76
Male	19
Female	17
**Clinical stage**	
I	17
II	5
III	14
**Histology**	
Adenocarcinoma	29
Squamous cell carcinoma	7
**Smoking history**	
Ever/current	11
No	25
**Adjuvant chemotherapy**	
Yes	22
No	14
**Adjuvant radiotherapy**	
Yes	12
No	24

Supplementary Table 2: The antibodies for immunoblotting

Antibodies	Source	Identifier	Dilution ratio
**p-STAT1**	Cell Signaling Technology	D4A7	1:1000
**p-STAT3**	Cell Signaling Technology	D3A7	1:1000
**p-STAT5**	Cell Signaling Technology	D47E7	1:1000
**p-STAT6**	Cell Signaling Technology	D8S9Y	1:1000
**STAT6**	Cell Signaling Technology	D3H4	1:1000
**IRF4**	Cell Signaling Technology	D9P5H	1:1000
**PU.1**	Cell Signaling Technology	9G7	1:1000
**Gata3**	Cell Signaling Technology	D13C9	1:1000
**p-p65**	Cell Signaling Technology	93H1	1:1000
**p65**	Cell Signaling Technology	D14E12	1:1000
**p-IKKα/β**	Cell Signaling Technology	16A6	1:1000
**IKKα**	Cell Signaling Technology	3G12	1:1000
**IKKβ**	Cell Signaling Technology	2C8	1:1000
**p-IκBα**	Cell Signaling Technology	14D4	1:1000
**IκBα**	Cell Signaling Technology	44D4	1:1000
**p-p38**	Cell Signaling Technology	D3F9	1:1000
**p38**	Cell Signaling Technology	D13E1	1:1000
**p-Akt**	Cell Signaling Technology	D9E	1:1000
**p-ERK**	Cell Signaling Technology	D13.14.4E	1:1000
**p-JNK**	Cell Signaling Technology	G9	1:1000
**p-Zap-70**	Cell Signaling Technology	65E4	1:1000
**Zap-70**	Cell Signaling Technology	D1C10E	1:1000
**β-actin**	Cell Signaling Technology	8H10D10	1:1000
**goat anti-mouse (HRP)**	Cell Signaling Technology	#7076	1:5000
**goat anti-rabbit (HRP)**	Cell Signaling Technology	#7074	1:5000
**p-PLCγ1**	Abcam	EP1898Y	1:3000
**PLCγ1**	Abcam	EP1898-7Y	1:3000
**p-NFAT1**	Abcam	ab200819	1:3000
**NFAT1**	Abcam	EPR2973	1:3000
**Fas**	Abcam	EPR5700	1:3000

Supplementary Table 3: Primers for real-time PCR

Gene	Primers
mActb **F**	5'-CGTTGACATCCGTAAAGACC-3'
mActb **R**	5'-AACAGTCCGCCTAGAAGCAC-3'
mIfng **F**	5'-GAGCTCATTGAATGCTTGGC-3'
mIfng **R**	5'-GCGTCATTGAATCACACCTG-3'
mIl4 **F**	5'-CGAGCTCACTCTCTGTGGTG-3'
mIl4 **R**	5'-TGAACGAGGTCACAGGAGAA-3'
mIl9 **F**	5'-AACAGTCCCTCCCTGTAGCA-3'
mIl9 **R**	5'-AAGGATGATCCACCGTCAAA-3'
mIl17a **F**	5'-TGAGCTTCCCAGATCACAGA-3'
mIl17a **R**	5'-TCCAGAAGGCCCTCAGACTA-3'
mFoxp3 **F**	5'-CTCGTCTGAAGGCAGAGTCA-3'
mFoxp3 **R**	5'-TGGCAGAGAGGTATTGAGGG-3'
mIrf4 **F**	5'-CAAAGCACAGAGTCACCTGG-3'
mIrf4 **R**	5'-TGCAAGCTCTTTGACACACA-3'
mSfpi1 **F**	5'-TGCAGCTCTGTGAAGTGGTT-3'
mSfpi1 **R**	5'-AGCGATGGAGAAAGCCATAG-3'
mTbx21 **F**	5'-CGTGGAGGTGAATGATGGA-3'
mTbx21 **R**	5'-TGGCAAAGGGGTTGTTGTCG-3'
mGata3 **F**	5'-AGGATGTCCCTGCTCTCCTT-3'
mGata3 **R**	5'-GCCTGCGGACTCTACCATAA-3'
mRorc **F**	5'-GGTGATAACCCCGTAGTGGA-3'
mRorc **R**	5'-CTGCAAAGAAGACCCACACC-3'
mEomes **F**	5'-CCACTGGATGAGGCAGGAGATT-3'
mEomes **R**	5'-GTCCTCTGTCACTTCCACGATG-3'
mGzmA **F**	5'-ACACGGTTGTTCCTCACTCAAGAC-3'
mGzmA **R**	5'-TCAATCAAAGCGCCAGCACAGATG-3'
mGzmC **F**	5'- GCAGAGGAGATAATCGGAGGC-3'
mGzmC **R**	5'-GCACGAATTTGTCTCGAACCA-3'
mGzmD **F**	5'-AGGTCCATCAATGACACTAAAGC-3'
mGzmD **R**	5'-TTGCATAGGCGAAAAGTCCAT-3'
mGzmE **F**	5'-CTGTGGAGGCTTCTTGGTTCA-3'
mGzmE **R**	5'-GATGTCACTGAAGAAGGCAGTG-3'
mGzmG **F**	5'-AAGGCCAAGAGAACTAAAGCTG-3'
mGzmG **R**	5'-CACACTGCACACATCCCCT-3'
hActb **F**	5'-CACCATTGGCAATGAGCGGTTC-3'
hActb **R**	5'-AGGTCTTTGCGGATGTCCACGT-3'
hIl9 **F**	5'-GACCAGTTGTCTCTGTTTGGGC-3'
hIl9 **R**	5'-TTTCACCCGACTGAAAATCAGTGG-3'
