# Supplementary material for: Fas signaling-mediated TH9 cell differentiation favors bowel inflammation and antitumor functions
Source: Nat Commun. 2019 Jul 2;10:2924. doi: 10.1038/s41467-019-10889-4 (PMC6606754; doi:10.1038/s41467-019-10889-4)
Supplement: Supplementary file 3 — Reporting Summary [file 41467_2019_10889_MOESM3_ESM.pdf]

nature research

Corresponding author(s): Zhijian Cai & Jianli Wang

Reporting Summary

Nature Research wishes to improve the reproducibility of the work that we publish. This form provides structure for consistency and transparency in reporting. For further information on Nature Research policies, see Authors & Referees and the Editorial Policy Checklist.

Statistical parameters

When statistical analyses are reported, confirm that the following items are present in the relevant location (e.g. figure legend, table legend, main text, or Methods section).

n/a	Confirmed	
☐	☒	The exact sample size (n) for each experimental group/condition, given as a discrete number and unit of measurement
☐	☒	An indication of whether measurements were taken from distinct samples or whether the same sample was measured repeatedly
☐	☒	The statistical test(s) used AND whether they are one- or two-sided<br>*Only common tests should be described solely by name; describe more complex techniques in the Methods section.*
☐	☒	A description of all covariates tested
☐	☒	A description of any assumptions or corrections, such as tests of normality and adjustment for multiple comparisons
☐	☒	A full description of the statistics including central tendency (e.g. means) or other basic estimates (e.g. regression coefficient) AND variation (e.g. standard deviation) or associated estimates of uncertainty (e.g. confidence intervals)
☐	☒	For null hypothesis testing, the test statistic (e.g. F, t, r) with confidence intervals, effect sizes, degrees of freedom and P value noted<br>*Give P values as exact values whenever suitable.*
☒	☐	For Bayesian analysis, information on the choice of priors and Markov chain Monte Carlo settings
☐	☒	For hierarchical and complex designs, identification of the appropriate level for tests and full reporting of outcomes
☐	☒	Estimates of effect sizes (e.g. Cohen's d, Pearson's r), indicating how they were calculated
☐	☒	Clearly defined error bars<br>*State explicitly what error bars represent (e.g. SD, SE, CI)*

Our web collection on statistics for biologists may be useful.

Software and code

Policy information about availability of computer code

Data collection	CytExpert experiment based software, Olympus Fluo View FV1000, Agilent 1100 series LC equipment
Data analysis	GraphPad Prism V7.0.1, FlowJo 10.4.2, FV10-ASW3.0 Viewer, LC 3D software, Microsoft Excel 2013

For manuscripts utilizing custom algorithms or software that are central to the research but not yet described in published literature, software must be made available to editors/reviewers upon request. We strongly encourage code deposition in a community repository (e.g. GitHub). See the Nature Research guidelines for submitting code & software for further information.

Data

Policy information about availability of data

All manuscripts must include a data availability statement. This statement should provide the following information, where applicable:

- Accession codes, unique identifiers, or web links for publicly available datasets
- A list of figures that have associated raw data
- A description of any restrictions on data availability

The authors declare that the data supporting the findings of this study are available within the paper and its supplementary information files.

Field-specific reporting

Please select the best fit for your research. If you are not sure, read the appropriate sections before making your selection.

☒ Life sciences ☐ Behavioural & social sciences ☐ Ecological, evolutionary & environmental sciences

For a reference copy of the document with all sections, see nature.com/authors/policies/ReportingSummary-flat.pdf

Life sciences study design

All studies must disclose on these points even when the disclosure is negative.

Sample size	Samples size for each experiment is indicated in the figures or corresponding figure legends. The number of samples assigned to each treatment was selected to provide sufficient statistical power to discern significant differences in different groups. This was based on prior experience with the experiment.
Data exclusions	No data were excluded from the analyses.
Replication	All replicates reported in the manuscript are biological replicates. All the statistics reported in the manuscript are based on at least 3 biologically independent replicates. All attempts to replicate the experiments were successful.
Randomization	Samples were randomized into different treatment groups.
Blinding	The assessment of clinical responses for patients was performed independently in a double-blind fashion. For mice studies, the experiments were performed in a blinded fashion when possible. Downstream analyses of mouse samples (immunofluorescence staining, flow cytometry and ELISA) were performed in a blinded fashion, which means that people performing the assays were not aware of the treatment groups until the data analyses were completed.

Behavioural & social sciences study design

All studies must disclose on these points even when the disclosure is negative.

Study description	*Briefly describe the study type including whether data are quantitative, qualitative, or mixed-methods (e.g. qualitative cross-sectional, quantitative experimental, mixed-methods case study).*
Research sample	*State the research sample (e.g. Harvard university undergraduates, villagers in rural India) and provide relevant demographic information (e.g. age, sex) and indicate whether the sample is representative. Provide a rationale for the study sample chosen. For studies involving existing datasets, please describe the dataset and source.*
Sampling strategy	*Describe the sampling procedure (e.g. random, snowball, stratified, convenience). Describe the statistical methods that were used to predetermine sample size OR if no sample-size calculation was performed, describe how sample sizes were chosen and provide a rationale for why these sample sizes are sufficient. For qualitative data, please indicate whether data saturation was considered, and what criteria were used to decide that no further sampling was needed.*
Data collection	*Provide details about the data collection procedure, including the instruments or devices used to record the data (e.g. pen and paper, computer, eye tracker, video or audio equipment) whether anyone was present besides the participant(s) and the researcher, and whether the researcher was blind to experimental condition and/or the study hypothesis during data collection.*
Timing	*Indicate the start and stop dates of data collection. If there is a gap between collection periods, state the dates for each sample cohort.*
Data exclusions	*If no data were excluded from the analyses, state so OR if data were excluded, provide the exact number of exclusions and the rationale behind them, indicating whether exclusion criteria were pre-established.*
Non-participation	*State how many participants dropped out/declined participation and the reason(s) given OR provide response rate OR state that no participants dropped out/declined participation.*
Randomization	*If participants were not allocated into experimental groups, state so OR describe how participants were allocated to groups, and if allocation was not random, describe how covariates were controlled.*

Ecological, evolutionary & environmental sciences study design

All studies must disclose on these points even when the disclosure is negative.

Study description	*Briefly describe the study. For quantitative data include treatment factors and interactions, design structure (e.g. factorial, nested, hierarchical), nature and number of experimental units and replicates.*

Research sample	*Describe the research sample (e.g. a group of tagged Passer domesticus, all Stenocereus thurberi within Organ Pipe Cactus National Monument), and provide a rationale for the sample choice. When relevant, describe the organism taxa, source, sex, age range and any manipulations. State what population the sample is meant to represent when applicable. For studies involving existing datasets, describe the data and its source.*
Sampling strategy	*Note the sampling procedure. Describe the statistical methods that were used to predetermine sample size OR if no sample-size calculation was performed, describe how sample sizes were chosen and provide a rationale for why these sample sizes are sufficient.*
Data collection	*Describe the data collection procedure, including who recorded the data and how.*
Timing and spatial scale	*Indicate the start and stop dates of data collection, noting the frequency and periodicity of sampling and providing a rationale for these choices. If there is a gap between collection periods, state the dates for each sample cohort. Specify the spatial scale from which the data are taken*
Data exclusions	*If no data were excluded from the analyses, state so OR if data were excluded, describe the exclusions and the rationale behind them, indicating whether exclusion criteria were pre-established.*
Reproducibility	*Describe the measures taken to verify the reproducibility of experimental findings. For each experiment, note whether any attempts to repeat the experiment failed OR state that all attempts to repeat the experiment were successful.*
Randomization	*Describe how samples/organisms/participants were allocated into groups. If allocation was not random, describe how covariates were controlled. If this is not relevant to your study, explain why.*
Blinding	*Describe the extent of blinding used during data acquisition and analysis. If blinding was not possible, describe why OR explain why blinding was not relevant to your study.*

Did the study involve field work? ☐ Yes ☐ No

Field work, collection and transport

Field conditions	*Describe the study conditions for field work, providing relevant parameters (e.g. temperature, rainfall).*
Location	*State the location of the sampling or experiment, providing relevant parameters (e.g. latitude and longitude, elevation, water depth).*
Access and import/export	*Describe the efforts you have made to access habitats and to collect and import/export your samples in a responsible manner and in compliance with local, national and international laws, noting any permits that were obtained (give the name of the issuing authority, the date of issue, and any identifying information).*
Disturbance	*Describe any disturbance caused by the study and how it was minimized.*

Reporting for specific materials, systems and methods

Materials & experimental systems

n/a	Involved in the study	
☐	☒	Unique biological materials
☐	☒	Antibodies
☐	☒	Eukaryotic cell lines
☐	☐	Palaeontology
☐	☒	Animals and other organisms
☐	☒	Human research participants

Methods

n/a	Involved in the study	
☐	☐	ChIP-seq
☐	☒	Flow cytometry
☐	☐	MRI-based neuroimaging

Unique biological materials

Policy information about availability of materials

Obtaining unique materials	Il9r-/- mice were kindly provided by Dr. Jean-Christophe Renauld (Université Catholique de Louvain, France). B16F10-OVA and LLC-OVA were provided by Dr. Qibin Leng (University of Chinese Academy of Sciences) and Wei Yang (Southern Medical University), respectively. All other materials are commercially available.

Antibodies

Antibodies used	The following primary antibodies were used for western blotting. They are listed as antigen first, followed by dilution, host, supplier, catalog number and clone number as applicable.<br>1) p-STAT1, 1:1000, Rabbit, Cell Signaling Technology, #7649, clone D4A7;<br>2) p-STAT3, 1:1000, Rabbit, Cell Signaling Technology, #9145, clone D3A7;<br>3) p-STAT5, 1:1000, Rabbit, Cell Signaling Technology, #4322, clone D47E7;<br>4) p-STAT6, 1:1000, Rabbit, Cell Signaling Technology, #56554, clone D8S9Y;<br>5) STAT6, 1:1000, Rabbit, Cell Signaling Technology, #5397, clone D3H4;<br>6) IRF4, 1:1000, Rabbit, Cell Signaling Technology, #15106, clone D9P5H;<br>7) PU.1, 1:1000, Rabbit, Cell Signaling Technology, #2258, clone 9G7;<br>8) Gata3, 1:1000, Rabbit, Cell Signaling Technology, #5852, clone D13C9;<br>9) p-p65, 1:1000, Rabbit, Cell Signaling Technology, #3033, clone 93H1;<br>10) p65, 1:1000, Rabbit, Cell Signaling Technology, #8242, clone D14E12;<br>11) p-IKKα/β, 1:1000, Rabbit, Cell Signaling Technology, #2697, clone 16A6,<br>12) IKKα, 1:1000, Mouse, Cell Signaling Technology, #11930, clone 3G12;<br>13) IKKβ, 1:1000, Rabbit, Cell Signaling Technology, #2370, clone 2C8;<br>14) p-IκBα, 1:1000, Rabbit, Cell Signaling Technology, #2859, clone 14D4<br>15) IκBα, 1:1000, Rabbit, Cell Signaling Technology, #4812, clone 44D4;<br>16) p-p38, 1:1000, Rabbit, Cell Signaling Technology, #11930, clone D3F9;<br>17) p38, 1:1000, Rabbit, Cell Signaling Technology, #8690, clone D13E1<br>18) p-Akt, 1:1000, Rabbit, Cell Signaling Technology, #4060, clone D9E;<br>19) p-ERK, 1:1000, Rabbit, Cell Signaling Technology, #4370, clone D13.14.4E;<br>20) p-JNK, 1:1000, Mouse, Cell Signaling Technology, #9255, clone G9;<br>21) β-actin, 1:1000, Mouse, Cell Signaling Technology, #3700, clone 8H10D10;<br>22) p-Zap70, 1:1000, Rabbit, Cell Signaling Technology, #2717, clone 65E4;<br>23) Zap70, 1:1000, Rabbit, Cell Signaling Technology, #3165, clone D1C10E;<br>24) p-PLCγ1, 1:3000, Rabbit, Abcam, ab76031, clone EP1898Y;<br>25) PLCγ1, 1:3000, Rabbit, Abcam, ab76155, clone EP1898-7Y;<br>26) p-NFAT1, 1:3000, Rabbit, Abcam, ab200819, clone N/A;<br>27) NFAT1, 1:3000, Rabbit, Abcam, ab92490, clone EPR2973;<br>28) Fas, 1:3000, Rabbit, Abcam, ab133619, clone EPR5700;<br>29) Secondary antibodies horseradish peroxidase-conjugated polyclonal goat anti-mouse, 1:1000, Goat, Cell Signaling Technology, #7076, clone N/A;<br>30) Secondary antibodies horseradish peroxidase-conjugated polyclonal goat anti-rabbit, 1:1000, Goat, Cell Signaling Technology, #7074, clone N/A;<br>The following primary antibodies were used for immunofluorescence. They are listed as antigen first, followed by dilution, host, supplier, catalog number and clone number as applicable.<br>31) Anti-Fas, 1:200, Mouse, Cell Signaling Technology, #8023, clone 4C3;<br>1) anti-PKCβ1, 1:200, Rabbit, Abcam, ab136971, clone A10-F;<br>2) anti-PKCβ2, 1:200, Rabbit, Abcam, ab184746, clone EPR18104;<br>3) anti-Fas antibody, 1:200, Rabbit, Abcam, ab133619, clone EPR5700;<br>4) anti-IL-9 antibody, 1:200, Rabbit, Abcam, ab181397, clone N/A; Cat#7649; RRID:<br>The following primary antibodies were used for flow cytometry. They are listed as antigen first, followed by dilution, host, supplier, catalog number and clone/lot number as applicable.<br>1) fixable viability dye eFluorTM 450, 1:500, N/A, eBioscience, #65-0863-14, N/A;<br>2) PE anti-CD4, 1:500, Rat, eBioscience, #12-0041-81, clone GK1.5;<br>3) APC anti-IFN-γ, 1:500, Rat, eBioscience, #17-7311-81, clone XMG1.2;<br>4) APC anti-IL-4, 1:500, Rat, eBioscience, #17-7041-81, clone 11B11;<br>5) APC anti-IL-9, 1:500, Rat, eBioscience, #50-8091-81, clone RM9A4;<br>6) APC anti-IL-17A, 1:500, Rat, eBioscience, #17-7177-81, clone eBio17B7;<br>7) APC anti-Foxp3, 1:500, Rat, eBioscience, #15-5773-81, clone FJK-16s;<br>8) PE anti-CD4, 1:500, Mouse, eBioscience, #12-0049-41, clone RPA-T4;<br>9) APC anti-IL-9, 1:500, Mouse, eBioscience, #MA5-23679, clone 623153;<br>10) CellTrace TM CFSE Cell Proliferation kit, N/A, N/A, Thermo Fisher, C34554, N/A;<br>11) Annexin V-FITC/PI, 1:200, N/A, MultiSciences, 70-AP101-100, N/A
Validation	All antibodies were purchased from commercial companies, and validated by the data sheets of the manufacturer or citations.

Eukaryotic cell lines

Policy information about cell lines

Cell line source(s)	Murine B16F10 tumor cells and HEK293 cells were obtained from the American Type Culture Collection (Manassas, VA, USA). B16F10-OVA and LLC-OVA were provided by Dr. Qibin Leng (University of Chinese Academy of Sciences, Shanghai, China) and Wei Yang (Southern Medical University, Guangzhou, Guangdong, China), respectively.
Authentication	All cell lines presented in this study were authenticated by DNA fingerprinting.
Mycoplasma contamination	All cells were routinely tested for mycoplasma contamination using a Mycoplasma Detection Kit (Lonza) and were found to be negative.

Commonly misidentified lines (See ICLAC register)	No commonly misidentified cell lines were used.

Palaeontology

Specimen provenance	*Provide provenance information for specimens and describe permits that were obtained for the work (including the name of the issuing authority, the date of issue, and any identifying information).*
Specimen deposition	*Indicate where the specimens have been deposited to permit free access by other researchers.*
Dating methods	*If new dates are provided, describe how they were obtained (e.g. collection, storage, sample pretreatment and measurement), where they were obtained (i.e. lab name), the calibration program and the protocol for quality assurance OR state that no new dates are provided.*

☐ Tick this box to confirm that the raw and calibrated dates are available in the paper or in Supplementary Information.

Animals and other organisms

Policy information about studies involving animals; ARRIVE guidelines recommended for reporting animal research

Laboratory animals	Female C57BL/6J (6-8-week old) mice and BALB/C-nu/nu mice were purchased from Joint Ventures Sipper BK Experimental Animal Co (Shanghai, China). Faslpr and Faslgld mice were purchased from the Jackson Laboratory (Farmington, CT, USA). Il9r-/- mice were kindly provided by Dr. Lionel Apetoh (Université de Bourgogne, Dijon, Bourgone, France). All female mice (6-8-week old) were used for this study. Mice were housed in a specific pathogen-free facility, and the experimental protocols were approved by the Animal Care and Use Committee of the School of Medicine, Zhejiang University.
Wild animals	The study did not involve wild animals.
Field-collected samples	The study did not involve field-collected samples.

Human research participants

Policy information about studies involving human research participants

Population characteristics	Tumor tissues of 36 individual patients with non-small cell lung carcinoma: 19 male, 17 female; age range 41-76 years; 10 stage IA, 2 stage IIA, 14 stage IIIA, 7 stage IB, 3 stage IIB. Whole blood of 11healthy donors: 7 male, 4 female; age range 24-32 years.
Recruitment	36 cancer patients with non-small cell lung carcinoma were recruited. Self-selection bias or other biases did not present in this study.

ChIP-seq

Data deposition

☐ Confirm that both raw and final processed data have been deposited in a public database such as GEO.

☐ Confirm that you have deposited or provided access to graph files (e.g. BED files) for the called peaks.

Data access links<br>*May remain private before publication.*	*For "Initial submission" or "Revised version" documents, provide reviewer access links. For your "Final submission" document, provide a link to the deposited data.*
Files in database submission	*Provide a list of all files available in the database submission.*
Genome browser session (e.g. UCSC)	*Provide a link to an anonymized genome browser session for "Initial submission" and "Revised version" documents only, to enable peer review. Write "no longer applicable" for "Final submission" documents.*

Methodology

Replicates	*Describe the experimental replicates, specifying number, type and replicate agreement.*
Sequencing depth	*Describe the sequencing depth for each experiment, providing the total number of reads, uniquely mapped reads, length of reads and whether they were paired- or single-end.*
Antibodies	*Describe the antibodies used for the ChIP-seq experiments; as applicable, provide supplier name, catalog number, clone name, and lot number.*
Peak calling parameters	*Specify the command line program and parameters used for read mapping and peak calling, including the ChIP, control and index files used.*
Data quality	*Describe the methods used to ensure data quality in full detail, including how many peaks are at FDR 5% and above 5-fold enrichment.*

Software | *Describe the software used to collect and analyze the ChIP-seq data. For custom code that has been deposited into a community repository, provide accession details.*

Flow Cytometry

Plots

Confirm that:

☒ The axis labels state the marker and fluorochrome used (e.g. CD4-FITC).

☒ The axis scales are clearly visible. Include numbers along axes only for bottom left plot of group (a 'group' is an analysis of identical markers).

☒ All plots are contour plots with outliers or pseudocolor plots.

☒ A numerical value for number of cells or percentage (with statistics) is provided.

Methodology

Sample preparation

For intracellular staining, Naïve CD4+CD62LhiCD44lo T cells were sorted from WT, Faslpr and Fasgld mice and differentiated into TH0, TH1, TH2, TH9, TH17 and Treg cells in the presence of plate-bound anti-CD3 and anti-CD28 antibodies. Naïve CD4+CD62LhiCD44lo T cells were sorted from WT mice differentiated into TH9 in the presence of 10 μg/ml ISO or antibodies against Jo2, or human naïve CD4+CD45RA+CD45RO- T cells were stimulated with 10 μg/ml ISO or antibodies against human Fas (anti-Fas), with or without the p38 inhibitor SB203580 at 0.4 μM. In some experiments, z-VAD-fmk (1 μM), BAY 11-7082 (0.4 μM), LY2409881 (0.5 μM), enzastaurin (0.5 μM), Go 6983 (0.01 μM), 2-APB (10 μM,), Xestospongin C (10 μM), U73122 (0.1 μM), manoalide (10 μM), SB203580 (0.4 μM,), FK506 (5 pM) or INCA-6 (50 nM) was added at the beginning of culture. Cells were cultured for 3 days, restimulated for 1 additional day and then stimulated for 4 h at 37°C in RPMI-1640 medium containing PMA (50 ng/ml) and ionomycin (1 μg/ml). After staining for surface markers, cells were fixed and permeabilized according to the manufacturer's instructions (Cytofix/Cytoperm Kit, Thermo Fisher Scientific) and then stained for intracellular products. The following monoclonal antibodies were used for flow cytometric analyses: fixable viability dye eFluorTM 450 or phycoerythrin-conjugated anti-CD4, or allophycocyanin-conjugated IFN-γ, IL-4, IL-9, IL-17A and Foxp3.
TILs were prepared by enzymatice digestion, followed by Percoll (GE Healthcare) gradient purification and then stained for intracellular products. The following monoclonal antibodies were used for flow cytometric analyses: fixable viability dye eFluorTM 450, FITC-conjugated CD45, PE-conjugated CD4, PE-conjugated CD8 or APC-conjugated IFN-γ, IL-9, IL-17A and Foxp3.
For proliferation staining, Naïve T cells from WT mice with or without CFSE labeling were differentiated into TH9 cells in the presence of plate-bound anti-CD3 and anti-CD28 antibodies for 3 days.
For apoptosis staining, TH9 cells were stained with Annexin V and PI.
For sorting naive CD4+T cells from mouse, naive CD4+T cells were stained with fixable viability dye eFluorTM 450, APC-cy7-conjugated CD4, PE-conjugated CD62L and FITC-conjugated CD44.
To detect the role of Fas or FasL protein in TH9 differentiation, we over-expressed T cells with Fas-retroviral. The following monoclonal antibodies were used for flow cytometric analyses: fixable viability dye eFluorTM 450 or PE-conjugated anti-CD4, or APC-conjugated IL-9.

Instrument | BeckmanCoulter DxFLEX flow cytometer

Software | CytExpert experiment based software (BeckmanCoulter,Inc) was used to collect events, and FlowJo software (TreeStar) was used to analyze the data.

Cell population abundance | N/A

Gating strategy | Single cell gates based on FSC-H and FSC-A, and SSC-H and SSC-A were used to exclude non-singlets. A morphology gate based on FSC-A and SSC-A was used to exclude debrees. A live/dead cell gate based on fixable viability dye was used to exclude dead cells. For analyzing the positivity of IL-9 in cells, TH0 cells stained with IL-9 specific antibodies were used to define the background non-specific staining, and then a CD4+IL-9+ gate was used for cells stained with CD4 and IL-9 specific antibodies based on the background. For identifying TH1 cells, a CD4+IFN-γ+ gating strategy was used. For identifying TH2 cells, a CD4+IL-4+ gating strategy was used. For identifying TH17 cells, a CD4+IL-17A+ gating strategy was used. For identifying Treg cells, a CD4+Foxp3 gating strategy was used. For analyzing T cell proliferation using the CFSE dilution assay, divided cells showing diluted CFSE were gated by taking unstimulated CFSE-labeled cells and non-labeled cells as the controls, which showed the CFSE intensity of non-divided cells and auto-fluorescence of the cells, respectively. For analysing T cell apoptosis, T cells were labeled with Annexin V and PI.

☒ Tick this box to confirm that a figure exemplifying the gating strategy is provided in the Supplementary Information.

Magnetic resonance imaging

Experimental design

Design type | *Indicate task or resting state; event-related or block design.*

Design specifications | *Specify the number of blocks, trials or experimental units per session and/or subject, and specify the length of each trial or block (if trials are blocked) and interval between trials.*

Behavioral performance measures	*State number and/or type of variables recorded (e.g. correct button press, response time) and what statistics were used to establish that the subjects were performing the task as expected (e.g. mean, range, and/or standard deviation across subjects).*

Acquisition

Imaging type(s)	*Specify: functional, structural, diffusion, perfusion.*
Field strength	*Specify in Tesla*
Sequence & imaging parameters	*Specify the pulse sequence type (gradient echo, spin echo, etc.), imaging type (EPI, spiral, etc.), field of view, matrix size, slice thickness, orientation and TE/TR/flip angle.*
Area of acquisition	*State whether a whole brain scan was used OR define the area of acquisition, describing how the region was determined.*

Diffusion MRI ☐ Used ☐ Not used

Preprocessing

Preprocessing software	*Provide detail on software version and revision number and on specific parameters (model/functions, brain extraction, segmentation, smoothing kernel size, etc.).*
Normalization	*If data were normalized/standardized, describe the approach(es): specify linear or non-linear and define image types used for transformation OR indicate that data were not normalized and explain rationale for lack of normalization.*
Normalization template	*Describe the template used for normalization/transformation, specifying subject space or group standardized space (e.g. original Talairach, MNI305, ICBM152) OR indicate that the data were not normalized.*
Noise and artifact removal	*Describe your procedure(s) for artifact and structured noise removal, specifying motion parameters, tissue signals and physiological signals (heart rate, respiration).*
Volume censoring	*Define your software and/or method and criteria for volume censoring, and state the extent of such censoring.*

Statistical modeling & inference

Model type and settings	*Specify type (mass univariate, multivariate, RSA, predictive, etc.) and describe essential details of the model at the first and second levels (e.g. fixed, random or mixed effects; drift or auto-correlation).*
Effect(s) tested	*Define precise effect in terms of the task or stimulus conditions instead of psychological concepts and indicate whether ANOVA or factorial designs were used.*

Specify type of analysis: ☐ Whole brain ☐ ROI-based ☐ Both

Statistic type for inference (See Eklund et al. 2016)	*Specify voxel-wise or cluster-wise and report all relevant parameters for cluster-wise methods.*
Correction	*Describe the type of correction and how it is obtained for multiple comparisons (e.g. FWE, FDR, permutation or Monte Carlo).*

Models & analysis

n/a	Involved in the study
☐	☐ Functional and/or effective connectivity
☐	☐ Graph analysis
☐	☐ Multivariate modeling or predictive analysis

Functional and/or effective connectivity	*Report the measures of dependence used and the model details (e.g. Pearson correlation, partial correlation, mutual information).*
Graph analysis	*Report the dependent variable and connectivity measure, specifying weighted graph or binarized graph, subject- or group-level, and the global and/or node summaries used (e.g. clustering coefficient, efficiency, etc.).*
Multivariate modeling and predictive analysis	*Specify independent variables, features extraction and dimension reduction, model, training and evaluation metrics.*
