# Supplementary material for: Fas signaling-mediated TH9 cell differentiation favors bowel inflammation and antitumor functions
Source: Nat Commun. 2019 Jul 2;10:2924. doi: 10.1038/s41467-019-10889-4 (PMC6606754; doi:10.1038/s41467-019-10889-4)
Supplement: Supplementary file 4 — Description of Additional Supplementary Files [file 41467_2019_10889_MOESM4_ESM.pdf]

Description of Additional Supplementary Files

File Name: Supplementary Data1

Description: RNA-sequencing analysis of differentially expressed genes in WT-T_H9 and *Fas*^{lpr}-T_H9 in Fig. 2
